# Nanoscale Crystalline Sheets and Vesicles Assembled from Nonplanar Cyclic *π*-Conjugated Molecules

**DOI:** 10.34133/2019/1953926

**Published:** 2019-07-28

**Authors:** Huang Tang, Zhewei Gu, Haifeng Ding, Zhibo Li, Shiyan Xiao, Wei Wu, Xiqun Jiang

**Affiliations:** ^1^MOE Key Laboratory of High Performance Polymer Materials and Technolog, and Department of Polymer Science & Engineering, College of Chemistry & Chemical Engineering, Nanjing University, Nanjing, 210093, China; ^2^National Laboratory of Solid State Microstructures and Department of Physics, Nanjing University, Nanjing, 210093, China; ^3^School of Polymer Science and Engineering, Qingdao University of Science and Technology, Qingdao, China; ^4^CAS Key Laboratory of Soft Matter Chemistry and Department of Polymer Science and Engineering, University of Science and Technology of China, Hefei, 230026, China

## Abstract

A fundamental challenge in chemistry and materials science is to create new carbon nanomaterials by assembling structurally unique carbon building blocks, such as nonplanar *π*-conjugated cyclic molecules. However, self-assembly of such cyclic *π*-molecules to form organized nanostructures has been rarely explored despite intensive studies on their chemical synthesis. Here we synthesized a family of new cycloparaphenylenes and found that these fully hydrophobic and nonplanar cyclic *π*-molecules could self-assemble into structurally distinct two-dimensional crystalline multilayer nanosheets. Moreover, these crystalline multilayer nanosheets could overcome inherent rigidity to curve into closed crystalline vesicles in solution. These supramolecular assemblies show that the cyclic molecular scaffolds are homogeneously arranged on the surface of nanosheets and vesicles with their molecular isotropic x-y plane standing obliquely on the surface. These supramolecular architectures that combined exact crystalline order, orientation-specific arrangement of *π*-conjugated cycles, controllable morphology, uniform molecular pore, superior florescence quench ability, and photoluminescence are expected to give rise to a new class of functional materials displaying unique photonic, electronic, and biological functions.

## 1. Introduction

The creation of new carbon nanomaterials with novel structures by tailorable building blocks is one of the most important goals in materials, chemical, and physical sciences [1–3]. Currently, the *π*-conjugated nanocarbon materials, including spherical fullerenes, cylindrical carbon nanotubes, sheet-like graphenes, and polycyclic aromatic hydrocarbons, have shown extraordinary electronic and optical properties and significant applications in electronics, photonics, and biomedical areas [4–7]. This has further promoted the development of advanced *π*-conjugated carbon nanostructure with controllable architecture and chemical functionality based on a variety of top-down and bottom-up synthetic approaches [8, 9]. The controllable self-assembly of the *π*-conjugated building blocks via noncovalent interactions between adjacent units can meet the continuously increasing demands for high-performance optoelectronics and biomedical materials through rational supramolecular design, and it offers abundant opportunity to tailor device performance [10]. However, assembly of structurally uniform and atomically precise carbon building blocks to carbon nanomaterials remains a great challenge [11, 12]. This is in part due to the difficulty in the synthesis of pure carbon- or benzene-based building blocks with unique structures and also in part due to the lack of capability to organize these building blocks with highly sophisticated and precisely regulated strategy [13]. Generally, amphiphilic molecules having both hydrophilic and hydrophobic components, such as lipids, surfactants, and block copolymers, are mandatory for molecular self-assembly in solution to form various morphologic assemblies [14, 15]. The predominant driving forces for self-assembly are hydrophobic, electrostatic, and hydrogen-bond interactions. In few cases, the driving forces of molecular assembly are induced by crystallization of block copolymers where crystalline block serves as the lyophobic component and amorphous block as solvated corona to stabilize assemblies [16]. Such amphiphilic concept has also been extended to hydrophobic amphiphilicity for self-assembly of spherical fullerene derivatives [17]. In this case, the molecular structure of fullerene derivatives contains two mutually immiscible spherical fullerene (solvophobic component) and long alkyl chains (solvophilic component) parts, and an amphiphilic structure framework still keeps. Thus, a question arises whether nonamphiphilic molecules cannot form stable assembly in solution.

**Scheme 1:**
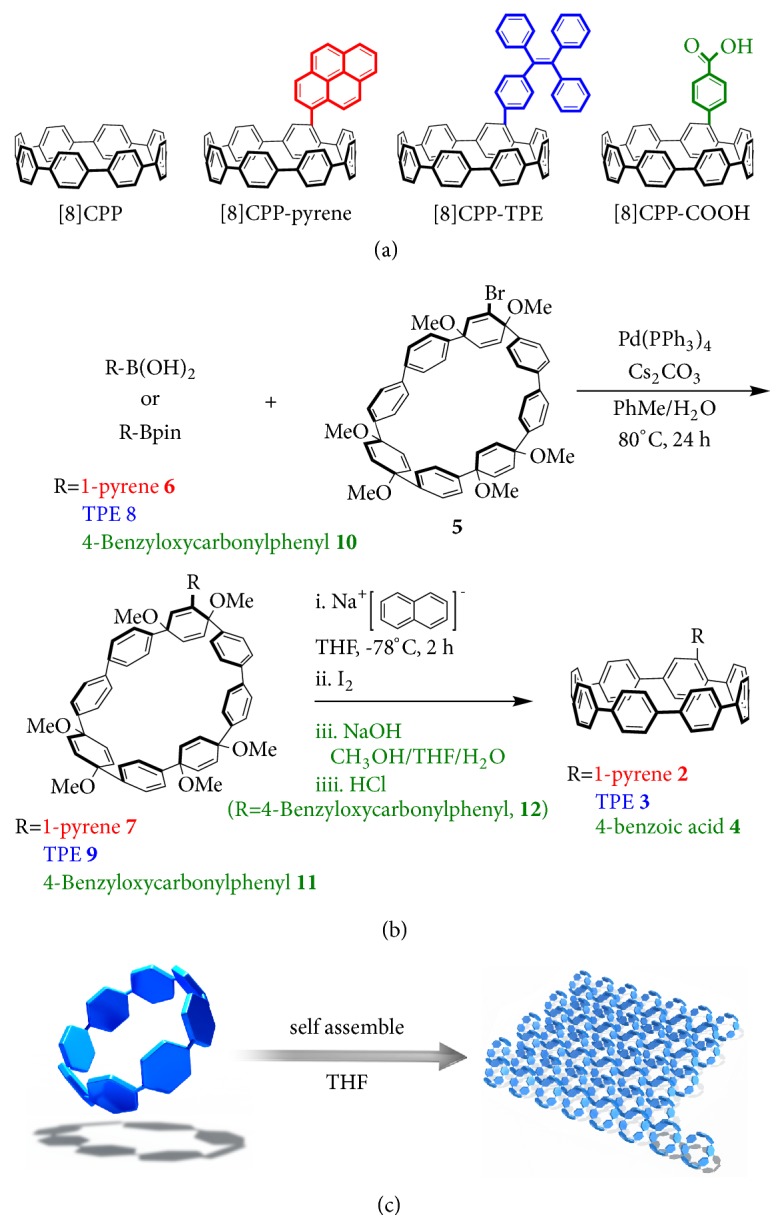
*Molecule design and self-assembly of CPPs.* (a) Chemical structures of [8]CPP, [8]CPP-pyrene, [8]CPP-TPE, and [8]CPP-COOH. (b) Synthesis route of [8]CPP-pyrene, [8]CPP-TPE, and [8]CPP-COOH. (c) Schematic illustration of the process of self-assembly of [8]CPPs into the nanosheets in THF.

[*n*]Cycloparaphenylenes ([*n*]CPPs) are a class of structurally unique hydrocarbon macrocycles consisting of a number of (*n*) benzene rings connected at the* para*-positions [18] and display unique photophysical [19, 20], redox [21, 22], porous [23], and host–guest [24, 25] properties. They are envisioned as the shortest cross-section of armchair carbon nanotube. Since first synthesized in 2008 [26], CPP molecules become one particularly promising class of functional molecules due to their novel character and are used as template for the bottom-up synthesis of carbon nanotubes [27]. The cyclic *π*-conjugated structure and cyclic shape-persistent scaffold of CPPs also make them fascinating building blocks to develop novel functional materials [28–31]. Recently, CPP films were prepared through Langmuir-Blodgett (LB) mechanism at an air-water interface [32]. Previous investigations suggested that planar and rigid *π*-conjugated organic molecules can self-assemble into 1D nanostructures through strong *π*–*π* stacking [33, 34], and some macrocycle molecules can self-assemble into different supramolecules [35, 36]. However, the self-assembly of unmodified cyclic CPPs to form organized nanostructures has not been achieved to date. The main difficulty lies in the fact that, unlike large planar macrocyclic aromatic molecules that favor coplanar arrangement and cofacial *π*–*π* stacking [14], CPPs have a nonplanar cyclic conjugated structure that weakens *π*–*π* interaction between CPP molecules [37]. Thus, the lack of molecular self-assembly of CPPs currently prevents the development of CPP-based carbon materials beyond molecular level. Herein we report the first example of using fully hydrophobic and nonplanar cyclic *π*-conjugated molecules as building blocks to self-assemble structurally distinct crystalline multilayer nanosheets and nanoscale crystalline multilayer vesicles in solution (Scheme 1(c)). It is demonstrated that fully hydrophobic nonplanar cyclic *π*-molecules can precisely stack together to form nanoscale crystalline assemblies with unique structural features in solution. The key driving force for the formation of nanostructured sheets and vesicles is the crystallization of nonplanar cyclic *π*-molecules in solution. These findings provide opportunities for deep understanding of the self-assembly mechanism for nonplanar cyclic *π*-conjugated molecules and facilitate the development of organic nanooptoelectronics and nanobiomedical devices.

## 2. Results

### 2.1. Design and Synthesis of [8]CPP and Its Derivatives

The synthesis and functionalization of CPP molecules have been a challenge due to the complex synthetic routes, low reactivity of benzene ring, and high strain energy [28, 30]. Initially, we designed and synthesized eight-membered cycloparaphenylene, i.e., pristine [8]CPP molecule (Scheme 1(a)), a known CPP compound. Then, the three different side groups were, respectively, incorporated into cyclic scaffold of [8]CPP to evaluate the effect of side group on CPPs properties. These three [8]CPP derivatives, pyrene-linked [8]CPP, tetraphenylethene-linked [8]CPP, and carboxyl-linked [8]CPP, were newly synthesized compounds, as shown in Scheme 1(a). The key to synthesize these new compounds was the use of bromosubstituted macrocycle as intermediate [28]. As shown in Scheme 1(b), 1-pyreneboronic acid** 6**, TPE-Bpin** 8**, and (4-benzyloxycarbonylphenyl) boronic acid** 10 **were connected to bromosubstituted macrocycle** 5** through Suzuki reaction, respectively. Then three different functionalized macrocycles, pyrene-substituted macrocycle** 7,** tetraphenylethene (TPE)-substituted macrocycle** 9**, and (4-benzyloxycarbonylphenyl)-substituted macrocycle** 11 **were obtained. After subjecting these different modified macrocycles to sodium naphthalenide at -78°C for 2 h and deprotection of (4-benzyloxycarbonylphenyl) [8]CPP** 12**, three different functionalized CPPs, pyrene-functionalized [8]CPP** 2,** TPE-functionalized [8]CPP** 3**, and carboxyl functionalized [8]CPP** 4**, were obtained. The synthesis and characterization of the resulting CPP molecules and their intermediates were described in detail in Experimental Procedures and Supplemental Information. In addition to chemical synthesis to incorporate side group into [8]CPP, these [8]CPP derivatives all were dual-nature molecules. For example, pyrene-functionalized [8]CPP derivative has incorporated both cyclic and planar structures in the molecule, and TPE-functionalized [8]CPP derivative possessed both cyclic and noncyclic structures whereas carboxyl functionalized [8]CPP molecule had both hydrophobic and hydrophilic moieties in its structure. It should also be pointed out that pyrene is a typical fluorescence dye and TPE has an aggregation-induced fluorescence emission (AIE) effect whereas carboxyl group bears negative charge and electrostatic effect. These CPP compounds were named to [8]CPP, [8]CPP-pyrene, [8]CPP-TPE, and [8]CPP-COOH, respectively. The spatial structures of [8]CPP and its derivatives were determined by density functional theory (DFT) methods using RB3LYP/6-31G(d). The data of [8]CPP were in good agreement with the results reported previously [21]. The morphology of [8]CPP was close to a circle, and the introduction of side chain made the circularity of [8]CPP macrocycle less regular. [8]CPP-pyrene and [8]CPP-TPE molecules had two different isomers and the main difference was that the pyrene or TPE group in the molecules had different angles with [8]CPP rings. Thus, the introduction of side chain had a slight effect on the circular structure of the [8]CPP ring ([Supplementary-material supplementary-material-1] and Tables [Supplementary-material supplementary-material-1]–[Supplementary-material supplementary-material-1]).

### 2.2. Spectral Properties of [8]CPP and Its Derivatives

**Figure 1:**
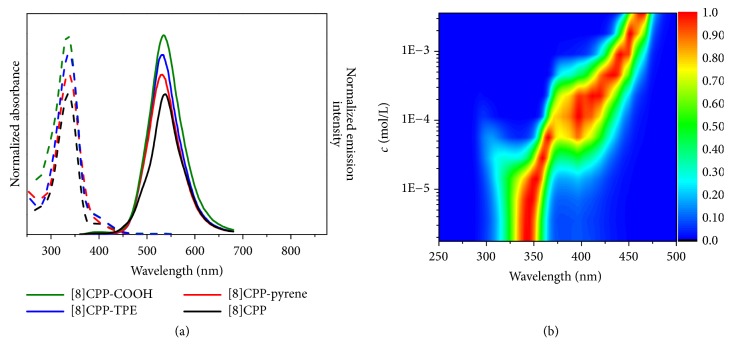
*Spectral Characterization of CPPs.* (a) Normalized UV−vis absorption (dashed line) and fluorescence emission spectra (solid line) of 10^–6^ M [8]CPP, [8]CPP-pyrene, [8]CPP-TPE, and [8]CPP-COOH in THF. The fluorescence emission spectra were obtained by exciting the samples at *λ*_ex_ = 340 nm. (b) Normalized color plots of the concentration-dependent fluorescence excitation spectra of [8]CPP between 10^–3^ M and 10^–6^ M in THF (*λ*_em_ = 540 nm).

The self-assembly behaviors of these CPPs in solution were initially investigated by UV−vis absorption and fluorescence spectra. The CPPs were dissolved in tetrahydrofuran (THF) and the concentration was chosen to be 10^−6^ M, dilute enough to decrease intermolecular interactions of CPP molecules in solution. All four CPPs showed the same maximum absorption peak at 340 nm in their absorption spectra regardless of their side groups even though the side groups of pyrene and TPE have strong UV absorption alone. Similarly, the photoluminescent emission (PL) spectra of [8]CPP derivatives exhibited the same maximum emission as that of pristine [8]CPP at 540 nm (Figure 1(a)). Notably, for pyrene as a typical fluorescence dye [38], its fluorescence signal was very strong in the synthesized macrocycle intermediate, [8]macrocycle-pyrene** 8** ([Supplementary-material supplementary-material-1]). However, its signal disappeared in the resulting product, [8]CPP-pyrene. Adding [8]CPP into pyrene in THF further confirmed the strong effect of fluorescence quench of [8]CPP ([Supplementary-material supplementary-material-1]). This result suggests that [8]CPP can quench the fluorescence of pyrene perfectly but keep itself fluorescence. Furthermore, it was found that [8]CPP could also quench the aggregation-induced fluorescence emission (AIE) of TPE [39], while AIE was greatly obvious in the form of [8]macrocycle-TPE** 10** ([Supplementary-material supplementary-material-1]). Adding [8]CPP into TPE in THF eliminated the AIE of TPE ([Supplementary-material supplementary-material-1]) but emitted [8]CPP fluorescence. The fluorescence quantum yields (Φ) of these [8]CPPs in THF were measured to be 0.10, 0.09, 0.09, and 0.10 for [8]CPP, [8]CPP-pyrene, [8]CPP-TPE, and [8]CPP-COOH, respectively ([Supplementary-material supplementary-material-1]). Thus, [8]CPP displays unique optical property. Unlike common fluorescence quenching agent, [8]CPP not only can effectively quench fluorescence of both pyrene and TPE but also maintains its luminescent characteristic.

Since spectral properties of *π*-conjugated molecules are sensitive to aggregation [14], we measured the excitation spectra of [8]CPP and its derivatives in THF with the concentrations range from 10^–6^ M to 10^–3^ M. When the concentration of CPP increased from 10^–6^ M to 10^–3^ M, the emission spectra of samples did not change ([Supplementary-material supplementary-material-1]). However, a gradually enlarging red shift of excitation spectra was found, together with a significant change in the spectral shape (Figure 1(b) and Figures [Supplementary-material supplementary-material-1] and [Supplementary-material supplementary-material-1]). In 3×10^–3^ M concentration, the excitation spectra of [8]CPP and its derivatives showed narrow peak at 460 nm. Compared with their absorption at 340 nm in dilute solution (10^−6^ M), a large red shift of about 120 nm in excitation spectra was observed in elevated concentration. This suggests that the aggregation of CPP molecules in the solutions of [8]CPP and its derivatives is induced by increasing solution concentrations. This result also implies that the packing and arrangement in the aggreagation state regulate the electronic properties of CPPs.

### 2.3. Morphology and Microstructure of CPP Supramolecular Assemblies

It is curious to probe the morphology and structural features of these CPP aggregates. To do so, cryogenic transmission electron microscopy (Cryo-TEM), regular transmission electron microscopy (TEM), atomic force microscopy (AFM), and high-resolution scanning tunneling microscopy (STM) were used to observe the samples in the solution and in the solid state, respectively. Cryo-TEM samples were prepared by dropping [8]CPPs solution on TEM grid and then plunged rapidly into liquid nitrogen or liquid ethane.

**Figure 2:**
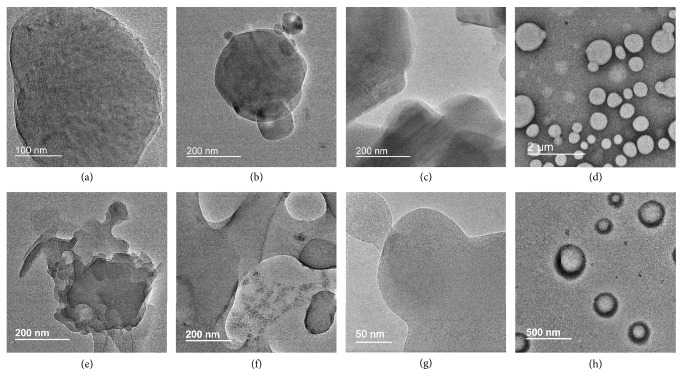
*Morphology Characterization of CPP aggregates by Electron Microscopic*. (a-d) Cryo-TEM images of assemblies formed by [8]CPP (a), [8]CPP-pyrene (b), [8]CPP-TPE (c), and [8]CPP-COOH (d) in THF solution (2×10^−5^ M). (e-h) Regular TEM images of assemblies formed by [8]CPP (e), [8]CPP-pyrene (f), [8]CPP-TPE (g), and [8]CPP-COOH (h) in THF (2×10^−5^ M), respectively.

The samples were imaged at approximately −170°C and no external staining was employed. It could be seen that [8]CPP, [8]CPP-pyrene, and [8]CPP-TPE formed the flat two-dimensional (2D) nanosheet morphology in THF (2×10^–5^ M), while [8]CPP-COOH formed the nanoscale vesicles (Figures 2(a)–2(d)). The dimensions of the nanosheets ranged from tens of nanometers to several hundred nanometers. The size of [8]CPP-COOH vesicles in THF ranged from about 300 nm to 1000 nm and the wall thickness is about 4–6 nm (Figure 2(d)). The self-assembled morphologies of [8]CPP and its derivatives were also verified by TEM at room temperature (Figures 2(e)–2(h)).

**Figure 3:**
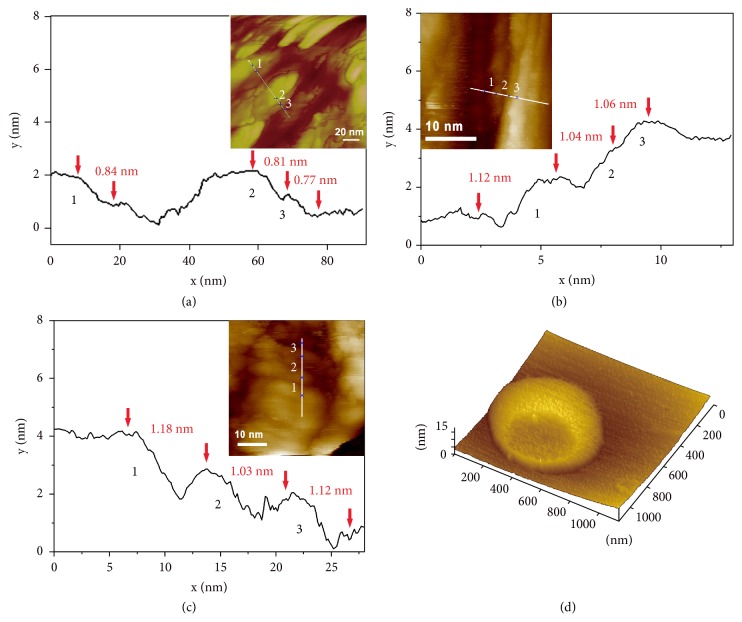
*Microstructure and Morphology Characterization of CPP aggregates*. (a) Thickness profile and AFM image (insert) of nanosheets formed by [8]CPP in THF solution. (b and c) Thickness profile and representative height-mode STM topography image (insert) of [8]CPP-pyrene (b) and [8]CPP-TPE (c), set point 20 pA, -1.0 V. (d) AFM image of supramolecular vesicles formed by [8]CPP-COOH in THF solution.

To evaluate the thickness of nanosheets, AFM and STM observations were performed. The single-layer thickness of the nanosheets was determined to be around 0.8 nm, 1.1 nm, and 1.1 nm for [8]CPP, [8]CPP-pyrene, and [8]CPP-TPE samples, respectively, indicating that the side group of [8]CPP had a significant effect on the film thickness (Figures 3(a)–3(c)). It is known that the diameter of the [8]CPP ring is about 1.1 nm [40]. The height of the [8]CPP ring, that is, the width of a benzene ring, is about 0.24 nm. The film thickness of [8]CPP sample was between the height and width of its molecule, implying that the ring is not perpendicular to the two-dimensional film plane or flat on the plane, but inclined to arrange at a certain angle. The collapse of the vesicle center in the AFM image of [8]CPP-COOH sample precipitated from THF was due to its hollow structure and demonstrates again its vesicle structure (Figure 3(d)). The size of the vesicle after collapse is about 800 nm, which is consistent with the phenomenon observed by cyro-TEM and TEM.

**Figure 4:**
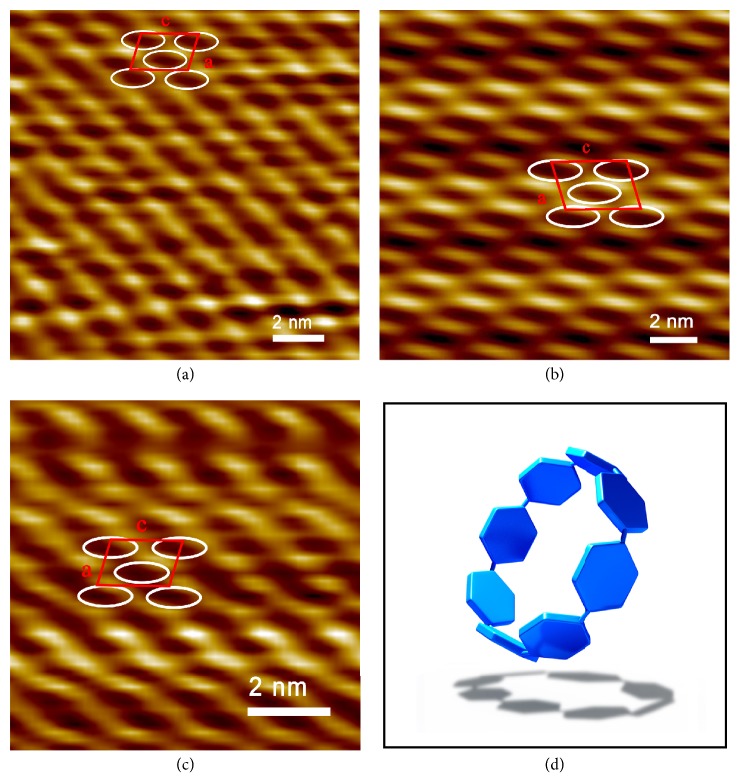
*Molecular Resolution STM Characterization of CPP nanosheet*. Molecular resolved STM topographic images of nanosheets formed by [8]CPP (a), [8]CPP-pyrene (b), and [8]CPP-TPE (c) in THF solution. (d) [8]CPP projected into an ellipse when it stood obliquely on the plane.

To gain insight into the subtle arrangement of CPP molecules in the nanosheets on molecular resolution, [8]CPP, [8]CPP-pyrene, and [8]CPP-TPE were deposited on the surface of highly oriented pyrolytic graphite (HOPG) from THF solution (2×10^−5^ M) and observed with high-resolution STM by using a high vacuum cryogenic STM system. Unexpectedly, in sharp contrast to a bright cyclic structure of flat-lying [8]CPPs in STM images observed at Au substrate surface in previous work [41], the bright short rods and dark elliptical cycles were clearly visualized in the molecular resolved STM images of [8]CPP, [8]CPP-pyrene, and [8]CPP-TPE three samples (Figures 4(a)–4(c)), suggesting again that the cyclic scaffold of individual [8]CPP molecules does not lie flat on the HOPG substrate. Instead, the CPP molecules stand up on the HOPG surface with certain tilt angle. In this way, the projection of cyclic CPP scaffolds on the substrate is ellipse, as shown in Figure 4(d).

This matches well with the observed single-layer thickness of 0.8 nm for [8]CPP nanosheets while its molecular diameter is 1.09 nm. The tilt angles between cyclic scaffold and substrate were calculated to be approximately 67° ± 3°, 68° ± 3°, and 68° ± 3°, for [8]CPP, [8]CPP-pyrene, and [8]CPP-TPE samples, respectively, based on the areas of ellipsoids and completely flat-lying CPPs.

**Figure 5:**
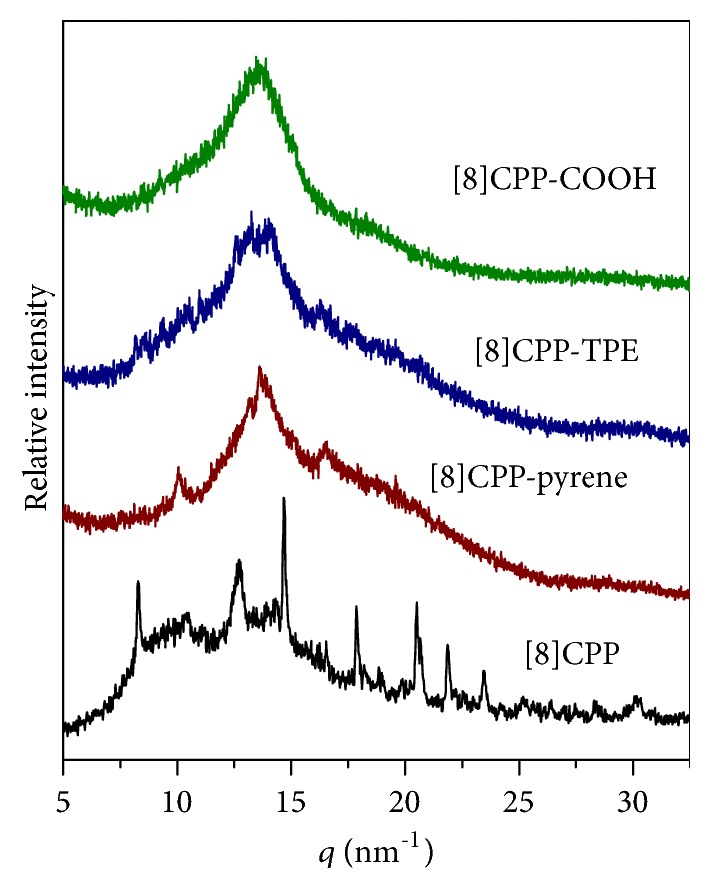
*XRD Characterization of CPP*. XRD patterns of [8]CPP, [8]CPP-pyrene, [8]CPP-TPE, and [8]CPP-COOH in powder states freeze-dried from THF solutions.

Next, we examined whether CPP molecules form a regular lattice in the nanosheets. For [8]CPP, its single crystal structure was previously reported [40], with a monoclinic lattice (a = 1.29 nm, b = 0.80 nm, c = 1.94 nm, and *β* = 105.4°). Interestingly, the STM image showed that the 2D unit cell of the [8]CPP nanosheets could be expressed by a lattice that was consistent with the a–c plane of its single-crystalline structure, with the measured lattice parameters in the two axes of 1.3 ± 0.1 nm and 1.9 ± 0.2 nm and the angle in between of 105° ± 3° (Figure 4(a)). Also, the packing distance perpendicular to the nanosheet was measured by AFM to be 0.8 ± 0.1 nm, which is in agreement with the length of “b” parameter in the single crystal. X-ray diffraction (XRD) analyses for the powder sample of [8]CPP nanosheets, which were obtained by freeze-drying from THF solutions at liquid-nitrogen temperature, further confirmed its crystal characteristic (Figure 5 and [Supplementary-material supplementary-material-1]). Together, based on the STM and AFM images as well as XRD measurement, it is concluded that the [8]CPP molecules pack into a crystalline structure in the nanosheets with the a–c plane of its unit structure at the surface of nanosheets. The lattice parameters are entirely similar to those of its single crystals. For [8]CPP-pyrene and [8]CPP-TPE nanosheets, STM imaging revealed similar unit cell to that of the [8]CPP, that is, two molecules in each cell and the angle between the two axes of 105° ± 3° (Figures 4(b) and 4(c)). XRD measurements for the powder samples of [8]CPP-pyrene and [8]CPP-TPE nanosheets suggest that [8]CPP-pyrene and [8]CPP-TPE molecules share similar crystalline structure with [8]CPP (Figure 5) but have varied unit cell parameters. These parameters could be measured from the STM images (Figures 3 and 4). For [8]CPP-pyrene, a = 2.1 ± 0.1 nm, b = 1.1 ± 0.1 nm, c = 2.9 ± 0.1, and *β* = 105° ± 3°. For [8]CPP-TPE, a = 1.3 ± 0.2 nm, b = 1.1 ± 0.1 nm, c = 1.9 ± 0.2, and *β* = 105° ± 3°. Compared to those of [8]CPP, only the values of lattice parameter “b” increase for [8]CPP-pyrene and [8]CPP-TPE samples, suggesting side group is arranged in the direction perpendicular to the surface of nanosheets.

On the other hand, [8]CPP-COOH did not form nanosheets in THF but assembled into vesicles (Figures 2(d), 2(h), and 3(d)). Therefore, the atomic revolution STM observation of [8]CPP-COOH vesicles could not be achieved but the XRD measurement of [8]CPP-COOH vesicles was done. The XRD pattern of [8]CPP-COOH vesicles was very similar to those of [8]CPP-pyrene and [8]CPP-TPE samples (Figure 5), indicating that the introduction of carboxyl side chains changed the morphology of CPP from nanosheet into vesicle, but it did not affect the microstructure of CPP greatly. STM data showed that the film thickness of [8]CPP derivatives was about 1.1 nm, and the vesicle thickness of [8]CPP-COOH was about 4-6 nm by freeze electron microscopy. This wall thickness of vesicles implies that [8]CPP-COOH vesicles have a multilayer-structural membrane.

## 3. Discussion

The current work reported the self-assembly of [8]CPP and its derivatives into nanoscale sheets and vesicles. So far, little work has been done about the self-assembly of CPP molecules and supramolecular properties. The reason arises from the unique structure of CPP molecules. Unlike isotropic spherical shape of fullerenes (C_60_), CPPs have an anisotropic cyclic shape. Although hexagonal C_60_ sheets could be prepared by crystallization and precipitation from appropriate solvents or at liquid-liquid interface [42], the solution self-assembly of C_60_ was only achieved by incorporating hydrophilic moieties or hydrophobic long alkyl chains into C_60_ scaffold [17, 43]. The former follows typical hydrophobic–hydrophilic amphiphilic molecular self-assembly, and the latter is called hydrophobic amphiphilic molecular self-assembly which utilizes different solvophobic or solvophilic character between spherical C_60_ scaffold and long alkyl chain [17]. In present work, we successfully achieved solution assembly of [8]CPP without aid of chemical modification and any additives such as surfactants and stabilizing agents although it has nonplanner anisotropic cyclic geometry and intrinsic hydrophobicity. It is the special cyclic conjugated structure of the CPPs that plays an important role in the novel self-assembly behaviors. As a control compound similar to the CPP structure but flat linear compound, unsubstituted linear oligoparaphenylene larger than sexiphenyl is practically insoluble and cannot self-assemble [44]. The self-assembly mechanism of [8]CPP is also significantly different from the common small molecules that have hydrophobic–hydrophilic or hydrophobic amphiphilic property. Based on STM observations and XRD measurements, the key driving force for the self-assembly of [8]CPPs in solution is the crystallization of [8]CPP molecules and its derivatives. The enthalpic gain from crystallization results in unique self-assembly behavior of [8]CPPs. Our self-assembly is also different from those crystallization-driven self-assemblies of diblock copolymers where one solvophilic block which extend into solvent phase is indispensable [16]. Thus, our self-assembly strategy allows up to 100% contents of [8]CPPs in assemblies. The crystallization drives [8]CPP, [8]CPP-pyrene, and [8]CPP-TPE to form the nanosheets and [8]CPP-COOH to the vesicles in THF. It is interesting that the cyclic scaffolds of CPPs are not arranged at the surface of nanosheets with their molecular isotropic x-y plane perpendicular to the surface but the x-y plane stands obliquely up on the surface with an tilt angle of about 67°~68°. This molecular packing fashion is favorable for the crystalline of [8]CPPs and formation of multilayer structure in the nanosheets further. If the x-y plane were perpendicular to the surface of nanosheets, a tubular morphology would be expected rather than sheet morphology [45]. If the x-y plane were parallel to the surface, a single-layer sheet or vesicle membrane will be obtained [46]. In fact, we observed a multilayer structure in the [8]CPP nanosheets and vesicle membrane. As the single crystal structure of [8]CPP that adopts a herringbone packing model, we believe that [8]CPP and its derivatives also adopt a herringbone arrangement pattern in the direction perpendicular to the nanosheet based on their lattice parameters measured. This is also in agreement with recent theoretic work [47, 48].

In summary, we had designed and synthesized four [8]CPP compounds with different chemical structures and demonstrated that these CPP compounds could self-assemble into crystalline multilayer nanosheets and vesicles. It is believed that crystallization of CPP building blocks plays a predominant role to drive the formation of CPP-based nanosheets and vesicles in solution. Particularly remarkable is that the cyclic CPP scaffolds pack together in a standing fashion with about a 67°−68° tilt angle. It was found that CPP moiety was a well effective fluorescence quenching agent that quenches dye's fluorescence but emits itself fluorescence. To comprehensively evaluate the nanosheet and vesicle performance, future efforts should be devoted to explore nanosheets' and vesicles' optoelectronic and biomedical performances. Further work in this direction will enable the next generation of functional carbon nanomaterials.

## 4. Materials and Methods

### 4.1. Materials and Instrumentation

All solvents for syntheses were dried by distillation under nitrogen prior to use. Tetrahydrofuran (THF) was distilled after reflux with sodium under nitrogen; dichloromethane (DCM) and N,N-dimethylformamide (DMF) were dried with CaH_2_. Other chemicals such as 1-pyreneboronic acid, tetraphenylethene (TPE), and (4-benzyloxycarbonylphenyl) boronic acid were obtained from commercial suppliers (Alfa, TCI, or J&K) and used without further purification. Moisture sensitive reactions were carried out under an inert atmosphere of nitrogen using standard syringe/septa technique.

High resolution mass spectrometry (HR-MS) analyses were carried out using MALDI-TOF-MS techniques. The matrix used for MALDI was a solution of 10 mg/ml of 7,7,8,8-tetracyanquinodimethane in THF with 1% silver trifluoroacetate as a promoter. Mass spectrometry of CPP and CPP derivatives could also be obtained without matrix. ^1^H NMR spectra and ^13^C NMR spectra were recorded at 300 MHz on a Bruker DXP-300 or at 400 MHz on a Bruker DQX-400. Chemical shifts for ^1^H NMR are shown in parts per million (ppm) relative to CDCl_3_ (*δ* 7.26 ppm). Chemical shifts for ^13^C NMR are expressed in ppm relative to CDCl_3_ (*δ* 77.0 ppm). UV absorption spectra were recorded on a Shimadzu UV-2401 spectrophotometer. Fluorescence emission and excitation spectra were measured on a Horiba FluoroMax-4 spectrofluorometer. Cryogenic transmission electronmicroscopy (cryo-TEM) images were captured on FEI T20 electron microscope. Transmission electron microscopy (TEM) images were captured on JEM-2011 electron microscope, JEM-1011 electron microscope, and FEI T20 electron microscope. Atomic force microscopy (AFM) was recorded on SEIKO SPI3800N and Veeco DiMultiMode V. STM measurements were conducted at ~78 K, with a home-built cryogenic (closed-cycle, cryostat-based) UHV STM system. XRD diffraction analysis was recorded on a Bruker XRD_D8 Discover.

### 4.2. Syntheses and Characterizations

#### 4.2.1. Synthesis of Pyrene-Substituted Macrocycle** 7**

A mixture of** bromo-substituted macrocycle 5 **(400 mg, 0.46 mmol), 1-pyreneboronic acid** 6** (168 mg, 0.68 mmol), Pd(PPh_3_)_4_ (52 mg, 0.048 mmol, 0.1 equiv), and Cs_2_CO_3_ (600 mg, 1.84 mmol, 4 equiv) was dissolved in 28 mL degassed Toluene/H_2_O (6:1) and stirred at 80°C for 24 h under nitrogen. The product was isolated as indicated in the Supplemental Information.

#### 4.2.2. Synthesis of [8]CPP-Pyrene** 2**

**Pyrene-substituted macrocycle 7** (240 mg, 0.240 mmol) was dissolved in 40 mL anhydrous THF under nitrogen and cooled down to −78°C. The freshly prepared sodium naphthalenide 2.0 mL (2.0 mmol, 1.0 M in THF) was added. The reaction was stirred for 2 h at −78°C; then 1.6 mL I_2_ (1 M solution in THF) was added. After warming up to room temperature, sodium thiosulfate saturated solution was carefully added to remove excess I_2_. The product [8]**CPP-pyrene 2** was isolated as indicated in the Supplemental Information.

#### 4.2.3. Synthesis of TPE-Substituted Macrocycle** 9**

A mixture of** bromo-substituted macrocycle 5** (400 mg, 0.46 mmol), TPE-Bpin** 8** (308 mg, 0.68 mmol), Pd(PPh_3_)_4_ (52 mg, 0.048 mmol, 0.1 equiv), and Cs_2_CO_3_ ( 600 mg, 1.84 mmol, 4 equiv) was dissolved in 28 mL degassed Toluene/H_2_O (6:1) and stirred at 80°C for 24 h under nitrogen atmosphere. The product was isolated as indicated in the Supplemental Information.

#### 4.2.4. Synthesis of [8]CPP-TPE** 3**

**TPE-substituted macrocycle 9** (280 mg, 0.28 mmol) was dissolved in 40 mL anhydrous THF under nitrogen and cooled down to −78°C. The freshly prepared sodium naphthalenide 2.4 mL (2.4 mmol, 1.0 M in THF) was added. After stirring for 2 h at −78°C, 2 mL I_2_ (1 M solution in THF) was added. The reaction was allowed to warm up to room temperature; then sodium thiosulfate saturated solution was carefully added to remove excess I_2_. The product [8]**CPP-TPE 3 **was isolated as indicated in the Supplemental Information.

#### 4.2.5. Synthesis of (4-Benzyloxycarbonylphenyl)-Substituted Macrocycle** 11**

**Bromo-substituted macrocycle 5** (400 mg, 0.46 mmol), (4-benzyloxycarbonylphenyl) boronic acid** 10 **(174 mg, 0.68 mmol), Pd(PPh_3_)_4_ (53 mg, 0.046 mmol, 0.1 equiv), and Cs_2_CO_3_ ( 600 mg, 1.84 mmol, 4 equiv) were dissolved in 28 mL degassed Toluene/H_2_O (6:1) and stirred at 80°C under nitrogen for 24 h. The product was isolated as indicated in the Supplemental Information.

#### 4.2.6. Synthesis of [8]CPP-COOH** 4**

**(4-Benzyloxycarbonylphenyl)-substituted macrocycle 11** (290 mg, 0.29 mmol) was dissolved in 80 mL anhydrous tetrahydrofuran under nitrogen and cooled to −78°C. The freshly prepared sodium naphthalenide 2.3 mL (2.3 mmol,1.0 M in THF) was added. The reaction was stirred for 2 h at −78°C; then 2.1 mL I_2_ (1 M solution in THF) was added. After the reaction mixture was warmed up to room temperature, sodium thiosulfate saturated solution was carefully added to remove excess I_2_. 40 mL water was added. After extraction with 3×40 mL dichloromethane, the combined organic phase was washed with 3×40 mL water and dried over sodium sulfate. After removing the solvent under vacuum, the crude yellow solid was used in next step directly without further purification.

To a stirred solution of this** 4-benzyloxycarbonylphenyl cycloparaphenylene 12** in a mixture of 50 mL CH_3_OH/ 50 mL THF was added 0.8 g NaOH in 10 mL H_2_O. The reaction mixture was allowed to stir for 18 h at room temperature. 0.1 M HCl was added to the reaction mixture until pH=2. The product [8]**CPP-COOH 4** was isolated as indicated in the Supplemental Information.

### 4.3. Quantum Yields Measurement

The quantum yields of CPPs were determined using the methods described by Ute Resch-Genger [49] using quinine (10% H_2_SO_4_) and anthracene (ethanol) as external standards. Excitation occurred at 340 nm. The fluorescence of [8]CPP and derivatives were integrated from 400 to 670 nm. The fluorescence of anthracene was integrated from 360 to 480 nm. The fluorescence of quinine was integrated from 400 to 600 nm.

### 4.4. Cryo-TEM Observation

Cryo-TEM samples were prepared by dropping CPPs solution on TEM grid and then plunged rapidly into liquid nitrogen or liquid ethane. The samples were imaged at about -170°C and no external staining was employed.

### 4.5. Freeze-Dried Method of CPP Tetrahydrofuran Solution

In a 50 ml Schlenk flask, the tetrahydrofuran solution of CPP is slowly dropped into liquid nitrogen; after tetrahydrofuran was frozen into solid, the flasks are transferred into a cold trap containing liquid nitrogen, using vacuum pump until freeze-dry of tetrahydrofuran.

## Data Availability

All data are available in the manuscript or supplementary materials.

## Supplementary Materials

Supplementary Materials Materials and Methods.* Figure S1*. Spatial structure of CPPs.* Figure S2.* Fluorescence spectra of [8]macrocycle-pyrene.* Figure S3.* Fluorescence quench of pyrene in the presence of [8]CPP.* Figure S4.* Aggregation-induced emission of [8]macrocycle-TPE.* Figure S5.* Fluorescence quench of TPE in the presence of [8]CPP.* Figure S6.* Quantum yield of CPPs.* Figure S7.* Fluorescence emission spectra of CPPs in concentrated THF solution.* Figure S8.* Fluorescence excitation spectra of CPPs at different concentration.* Figure S9*. Red-shift of CPP excitation spectra in different concentration.* Tables S1–S3.* Major bond lengths, dihedral angles for [8]CPP, [8]CPP-pyrene, and [8]CPP-TPE determined by DFT methods using RB3LYP/6-31G(d).* Table S4.* Crystal information of [8]CPP nanosheet powders freeze-dried from THF.* Figures S10–13*. MALDI-TOF spectra.* Figures S14–31*. ^1^H NMR and ^13^C NMR spectra.
