# Supplementary material for: Nanoscale Crystalline Sheets and Vesicles Assembled from Nonplanar Cyclic π-Conjugated Molecules
Source: Research (Wash D C). 2019 Jul 28;2019:1953926. doi: 10.34133/2019/1953926 (PMC6750094; doi:10.34133/2019/1953926)
Supplement: Supplementary Materials — Materials and Methods. Figure S1. Spatial structure of CPPs. Figure S2. Fluorescence spectra of [8]macrocycle-pyrene. Figure S3. Fluorescence quench of pyrene in the presence of [8]CPP. Figure S4. Aggregation-induced emission of [8]macrocycle-TPE. Figure S5. Fluorescence quench of TPE in the presence of [8]CPP. Figure S6. Quantum yield of CPPs. Figure S7. Fluorescence emission spectra of CPPs in concentrated THF solution. Figure S8. Fluorescence excitation spectra of CPPs at different concentration. Figure S9. Red-shift of CPP excitation spectra in different concentration. Tables S1–S3. Major bond lengths, dihedral angles for [8]CPP, [8]CPP-pyrene, and [8]CPP-TPE determined by DFT methods using RB3LYP/6-31G(d). Table S4. Crystal information of [8]CPP nanosheet powders freeze-dried from THF. Figures S10–13. MALDI-TOF spectra. Figures S14–31. 1H NMR and 13C NMR spectra. [file 1953926.f1.docx]

Supplementary Materials

**Nanoscale Crystalline Sheets and Vesicles Assembled from Non-Planar Cyclic π-Conjugated Molecules**

Huang Tang^1^, Zhewei Gu^1^, Haifeng Ding^2^, Zhibo Li^3^, Shiyan Xiao^4^, Wei Wu^1^, Xiqun Jiang*^,1^

^1^MOE Key Laboratory of High Performance Polymer Materials and Technology, and Department of Polymer Science & Engineering, College of Chemistry & Chemical Engineering, Nanjing University, Nanjing, 210093, China.

^2^National Laboratory of Solid State Microstructures and Department of Physics, Nanjing University, Nanjing, 210093, China.

^3^School of Polymer Science and Engineering, Qingdao University of Science and Technology, Qingdao, China.

^4^CAS Key Laboratory of Soft Matter Chemistry and Department of Polymer Science and Engineering, University of Science and Technology of China, Hefei, 230026, China.

* To whom correspondence should be addressed.

E-mail: jiangx@nju.edu.cn

**Table of content**

**1. Methods and Experiments**

1.1 Synthesis details

**2. Supplementary data of CPPs and their assemblies**

**3. Spectrum data of synthesized intermediates and resulting products**

3.1 MALDI-TOF spectra

3.2 ^1^H NMR and ^13^C NMR spectra

**4. References**

**1. Methods and Experiments**

**1.1 Synthesis**

**1**

[8]CPP **1** was prepared according to Ramesh Jasti's previously published procedures.[1] ^1^H NMR (400 MHz，CDCl_3_): δ(ppm) 7.48 (s, 32H). ^13^C NMR (100 MHz, CDCl_3_): δ(ppm) 127.44, 137.61. MALDI-TOF m/z calcd for C_48_H_32_ (M)^+^:608.25, Found: 608.2529. IR (neat): 817, 1023, 1083, 1260, 1483, 2854, 2925 cm^−1^.

**5**

The bromo-substituted macrocycle **5** used in this work was prepared according to Ramesh Jasti's previously published procedures.[2] ^1^H NMR (400 MHz, CDCl_3_): δ(ppm) 7.53-7.45 (m,14H, Ar), 7.35 (d, *J* = 7.7 Hz, 2H, Ar), 7.22 (t, *J* = 8.4 Hz, 2H, Ar), 7.08 (d, *J* = 8.5 Hz, 2H, Ar), 6.79 (d, *J* = 1.9 Hz, 1H, Vinyl-H), 6.26-6.23 (m, *2*H, Vinyl-H), 6.15-6.12 (overlap, 4H, Vinyl-H), 6.08–6.04 (overlap, 4H, Vinyl-H), 3.49–3.46 (overlap,12H, OMe), 3.40 (s, 6H, OMe). ^13^C NMR (100 MHz, CDCl_3_): δ(ppm) 143.35, 143.31, 143.02, 142.85, 140.54, 140.12, 139.47, 139.42, 138.69, 138.31, 134.55, 133.91, 133.73, 133.49, 133.34, 133.25, 132.97, 132.75, 132.60, 131.33, 128.90, 128.33, 128.11, 127.30, 127.19, 126.80, 126.32, 126.25, 125.94, 78.84, 74.64, 74.53, 74.04, 73.97, 52.42, 52.12, 51.93, 51.79. MALDI-TOF m/z calcd for C_53_H_47_BrO_5_ (M-OMe)^+^:842.26, Found: 841.621, 842.614. IR (neat): 821, 950, 1017, 1083, 1175, 1397, 1491, 2822, 2931 cm^−1^.


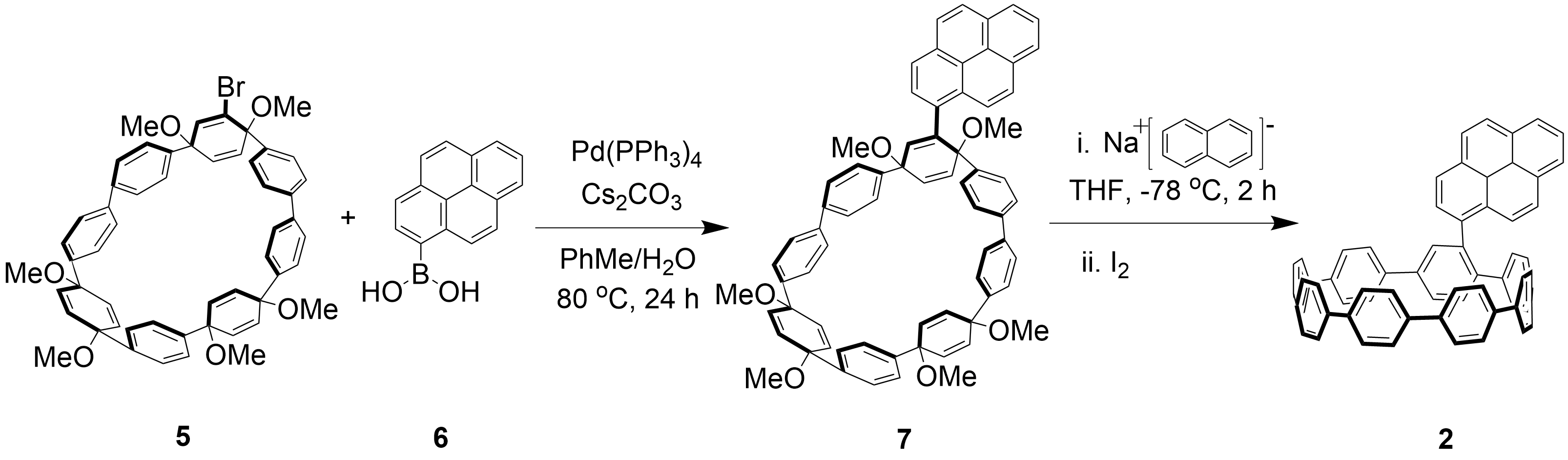

**7**

A mixture of bromo-substituted macrocycle **5** (400 mg, 0.46 mmol), 1-pyreneboronic acid **6** (168 mg, 0.68 mmol), Pd(PPh_3_)_4_ (52 mg, 0.048 mmol, 0.1 equiv) and Cs_2_CO_3_ (600 mg, 1.84 mmol, 4 equiv) were dissolved in 28 mL degassed Toluene/H_2_O (6:1) and stirred at 80 ^o^C for 24 h under nitrogen. The reaction was allowed to cool to room temperature, then 60 mL water was added. The aqueous phase was extracted with 3×60 mL dichloromethane and the combined organics were washed with 3×60 mL water and dried over anhydrous sodium sulfate. After removing the solvent under vacuum, the crude yellow solid was purified by by silica column chromatography using ethyl acetate/ hexane = 1:4. White solid of **7** (248 mg, 54%) was obtained. ^1^H NMR (300 MHz, CDCl_3_) :δ(ppm) 8.60 (s, 1H, Ar), 8.19–7.87 (overlap, 8H, Ar), 7.59–7.33 (overlap, 18H, Ar), 7.14 (d, *J* = 8.1 Hz, 2H, Vinyl-H), 6.80 (s, 1H, Vinyl-H), 6.58–6.43 (overlap, 2H, Vinyl-H), 6.21–6.00 (overlap, 8H, Vinyl-H), 3.74 (s, 3H, OMe), 3.51–3.42 (overlap, 12H, OMe), 3.31 (s, 3H, OMe). ^13^C NMR (75 MHz, CDCl_3_): δ(ppm) 143.37, 143.03, 142.93, 140.85, 140.47, 139.80, 139.60, 133.96, 133.64, 133.22, 132.77, 132.45, 131.41, 130.75, 130.40, 129.63, 129.21, 128.15, 127.58, 127.47, 127.22, 126.86, 126.30, 125.99, 125.78, 125.24, 79.90, 74.66, 74.54, 74.14, 52.57, 52.14, 51.88. MALDI-TOF m/z calcd for C_70_H_58_O_6_ (M)^+^: 994.42, Found: 994.726. IR (neat): 820, 949, 1016, 1080, 1174, 1490, 2929 cm^−1^.

The preparation of sodium naphthalenide (1.0 M in THF) was according to Ramesh Jasti's previously published procedures.^1^ Briefly, 768 mg naphthalene (6.00 mmol) was dissolved in 6 mL anhydrous THF and 207 mg sodium metal (9.00 mmol) was added under nitrogen. The reaction was stirred for 18 h at room temperature. After this time, a green solution of sodium naphthalenide (1.0 M in THF) was formed.

**2**

Pyrene-substituted macrocycle **7** (240 mg, 0.240 mmol) was dissolved in 40 mL anhydrous THF under nitrogen and cooled down to −78 ^o^C. The freshly prepared sodium naphthalenide 2.0 mL (2.0 mmol, 1.0 M in THF) was added. The reaction was stirred for 2 h at −78 ^o^C, then 1.6 mL I_2_ (1 M solution in THF) was added. After warming up to room temperature, sodium thiosulfate saturated solution was carefully added to remove excess I_2_. 40 mL water was added. After extraction with 3×40 mL dichloromethane, the combined organic phase was washed with 3×40 mL water and dried over sodium sulfate and concentrated in vacuo to deliver a yellow solid. This solid was purified by by silica column chromatography using DCM/hexane = 1:1. After removing all the solvents, a yellow solid (84 mg, 43%) was obtained. ^1^H NMR (400 MHz, CDCl_3_) :δ(ppm) 8.29–7.90 (overlap, 9H, Ar), 7.76–7.30 (overlap, 22H, Ar), 7.17–6.73 (overlap, 9H, Ar). ^13^C NMR (100 MHz, CDCl_3_): δ(ppm) 137.77(multiple overlapping peaks), 131.45, 127.47 (multiple overlapping peaks), 126.01. MALDI-TOF m/z calcd for C_64_H_40_ (M)^+^: 808.31, Found: 808.3513. IR (neat): 817, 1018, 1096, 1260, 1482, 2854, 2925 cm^−1^.


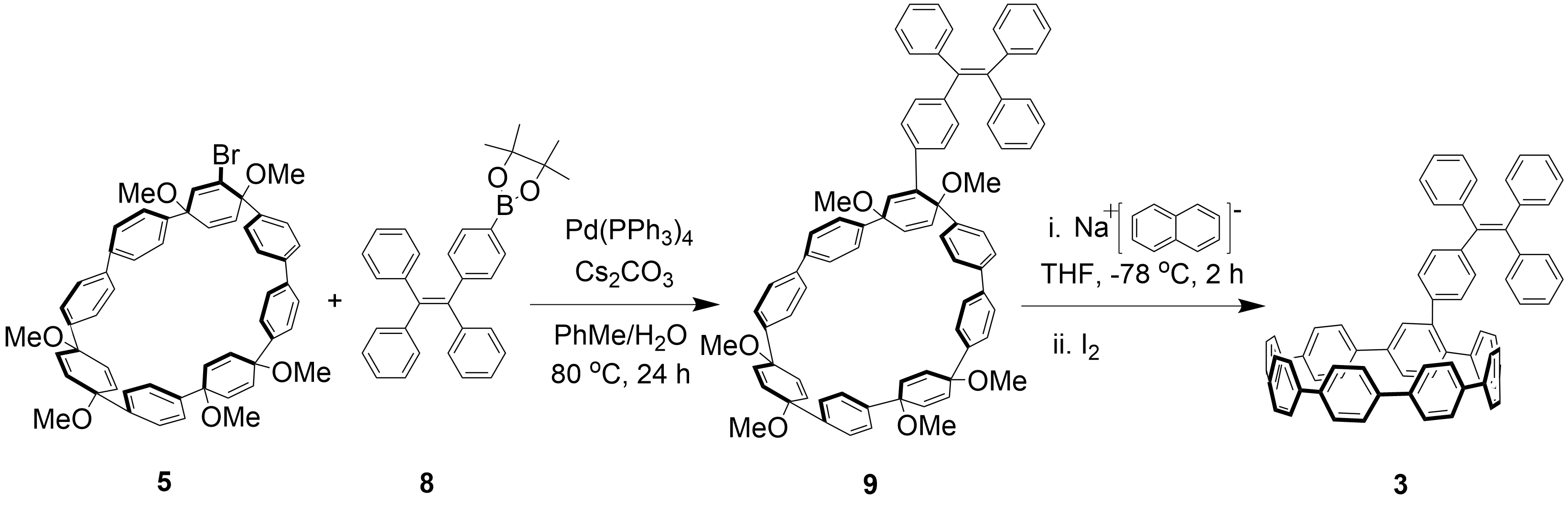

**8**

TPE-Bpin **8** was prepared according to Zhen Li's previously published procedures.[3] ^1^H NMR (300 MHz, CDCl_3_): δ(ppm) 7.56 (d, *J* = 8.2 Hz, 2H, Ar), 7.12–7.02 (m, 17H, Ar), 1.34 (s, 12H, CH_3_). ^13^C NMR (75 MHz, CDCl_3_): δ(ppm) 149.96, 143.81, 143.65, 141.55, 140.97, 135.42, 134.31, 133.31, 131.48, 130.87, 130.43, 129.79, 128.93, 127.82, 126.65, 125.60, 83.77, 25.92, 25.10, 24.24. MS-ESI m/z calcd for C_32_H_31_BO_2_ (M)^+^:458.24, Found: 458.15. IR (neat): 856, 1087, 1143, 1358, 2980 cm^−1^.

**9**

A mixture of bromo-substituted macrocycle **5** (400 mg, 0.46 mmol), TPE-Bpin **8** (308 mg, 0.68 mmol), Pd(PPh_3_)_4_ (52 mg, 0.048 mmol, 0.1 equiv) and Cs_2_CO_3_ ( 600 mg, 1.84 mmol, 4 equiv) were dissolved in 28 mL degassed Toluene/H_2_O (6:1) and stirred at 80 ^o^C for 24 h under nitrogen atmosphere. The reaction mixture was allowed to cool to room temperature, then 60 mL water was added. After extraction with 3×40 mL dichloromethane, the combined organics were washed with 3×40 mL water. The organic layer was evaporated under vacuum after drying with sodium sulfate. The crude yellow solid was purified by silica column chromatography using ethyl acetate/hexane = 1:4 obtaining the pure product as white solid (300 mg, 58%). ^1^H NMR (300 MHz, CDCl_3_): δ(ppm) 7.56–7.42 (overlap, 16H, Ar), 7.36–7.29 (overlap, 4H, Ar), 7.25–6.85 (overlap, 19H, Ar), 6.80 (d, *J* = 1.7 Hz, 1H, Vinyl-H), 6.26–6.24 (overlap, 2H, Vinyl-H), 6.17–6.12 (overlap, 4H, Vinyl-H), 6.09–6.04 (overlap, 4H, Vinyl-H), 3.49–3.47 (overlap, 12H, OMe), 3.41 (s, 6H, OMe). ^13^C NMR (75 MHz, CDCl_3_): δ(ppm) 143.64, 143.38, 143.08, 142.90, 140.60, 140.18, 139.52, 138.76, 138.37, 134.62, 133.79, 133.55, 133.37, 133.03, 132.83, 132.66, 132.44, 131.36, 128.16, 127.96, 127.62, 127.36, 127.11, 126.86, 126.45, 126.38, 126.31, 78.85, 74.75, 74.61, 74.09, 52.49, 52.18, 51.86, 51.25. MALDI-TOF m/z calcd for C_80_H_68_O_6_ (M)^+^: 1124.50, Found: 1124.963. IR(neat): 820, 951, 1016, 1083, 1261, 1491, 2822, 2927 cm^−1^.

**3**

TPE-substituted macrocycle **9** (280 mg, 0.28 mmol) was dissolved in 40 mL anhydrous THF under nitrogen and cooled down to −78 ^o^C. The freshly prepared sodium naphthalenide 2.4 mL (2.4 mmol, 1.0 M in THF) was added. After stirring for 2 h at −78 ^o^C, 2 mL I_2_ (1 M solution in THF) was added. The reaction was allowed to warm up to room temperature, then sodium thiosulfate saturated solution was carefully added to remove excess I_2_ and 40 mL water was added. After extraction with 3×40 mL dichloromethane, the combined organic phase was washed with 3×40 mL water and dried over sodium sulfate. The organics were then concentrated under vacuum to deliver a yellow solid which could be further purified by silica column chromatography using DCM/hexane = 1:1. **3** was obtained as yellow solid (120 mg, 52%). ^1^H NMR (300 MHz, CDCl_3_): δ(ppm) 7.52–7.42 (overlap, 31H, Ar), 7.12–7.01 (overlap, 19H, Ar). ^13^C NMR (100 MHz, CDCl_3_): δ(ppm) 137.64 (multiple overlapping peaks), 131.75, 131.35, 129.15, 128.57, 128.20, 127.47 (multiple overlapping peaks), 126.51. MALDI-TOF m/z calcd for C_74_H_50_ (M)^+^: 938.39, Found: 938.4478. IR (neat):817, 1019, 1094, 1260, 1483, 2854, 2925 cm^−1^.


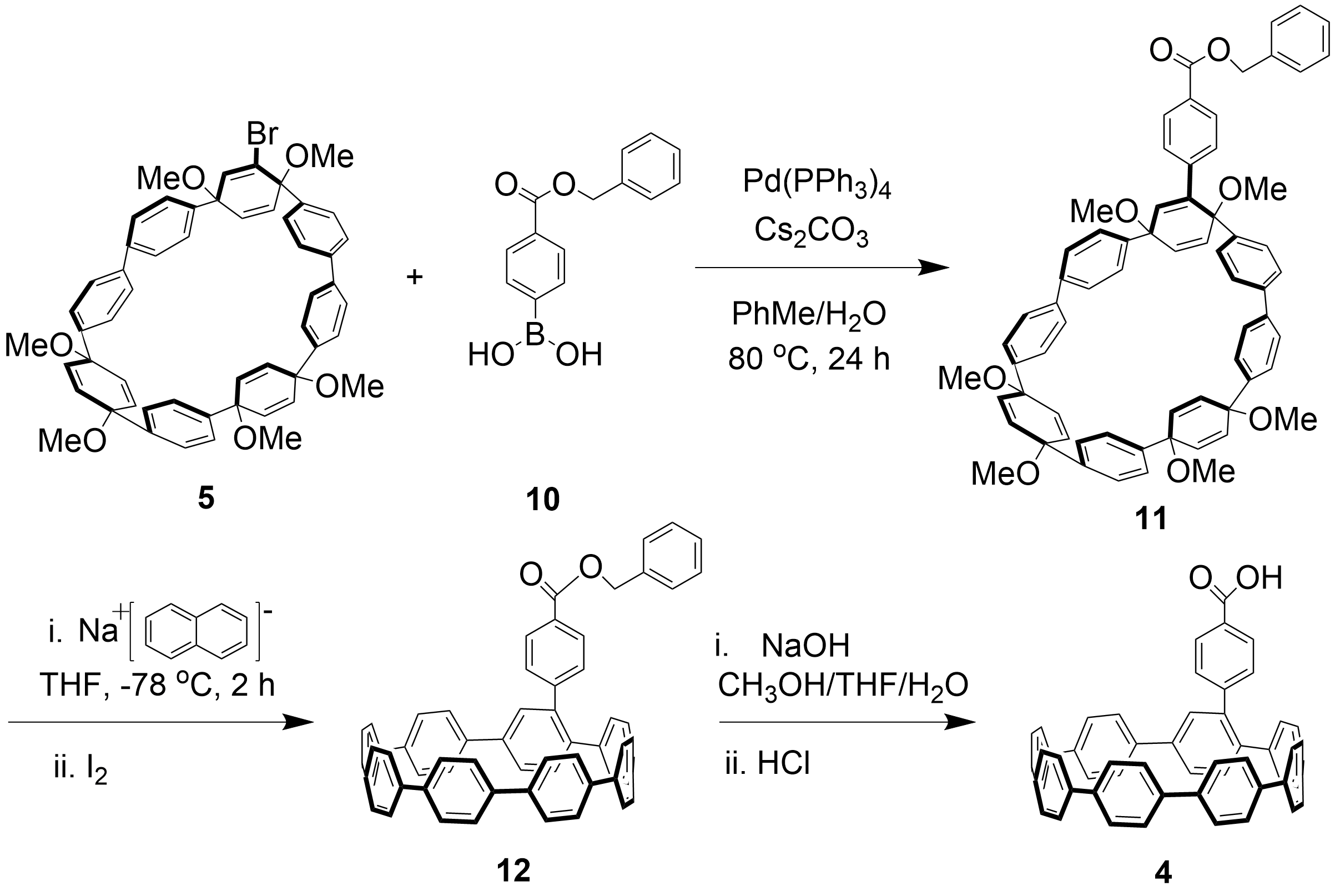

**11**

Bromo-substituted macrocycle **5** (400 mg, 0.46 mmol), (4-Benzyloxycarbonylphenyl) boronic acid **10** (174 mg, 0.68 mmol), Pd(PPh_3_)_4_ (53 mg, 0.046 mmol, 0.1 equiv) and Cs_2_CO_3_ ( 600 mg, 1.84 mmol, 4 equiv) were dissolved in 28 mL degassed Toluene/H_2_O (6:1) and stirred at 80 ^o^C under nitrogen for 24 h. After cooling down to room temperature, 60 mL water was added. After extraction with 3×60 mL dichloromethane_,_ the combined organic phase was washed with 3×60 mL water and dried over anhydrous sodium sulfate. After removing the solvent under vacuum, the crude yellow solid was further purified by passing the mixture through a short plug of silica gel using ethyl acetate/hexane = 1:4 as the mobile phase. Concentration of the eluent then delivered a white solid (290 mg, 63%). ^1^H NMR (400 MHz, CDCl_3_): δ(ppm) 7.92 (d, *J* = 8.5 Hz, 2H,Ar), 7.27−7.54 (overlap, 25H, Ar), 7.09 (d, *J* = 8.3 Hz, 2H, Ar), 6.86 (d, *J* = 1.7 Hz, 1H, Vinyl-H), 6.31 – 6.21 (m, 2H, Vinyl-H), 6.18 – 6.12 (overlap, 4H, Vinyl-H), 6.09 – 6.04 (overlap, 4H, Vinyl-H), 5.31 (s, 2H,CH_2_), 3.53-3.41 (overlap, 15H, OMe), 3.18 (s, 3H, OMe). ^13^C NMR (100 MHz, CDCl_3_): δ(ppm) 166.17, 143.37, 142.97, 142.93, 142.12, 140.62, 140.37, 139.82, 139.64, 139.48, 139.16, 137.95, 137.45, 136.07, 134.00, 133.55, 132.90, 132.44, 129.41, 129.14, 128.80, 128.56, 128.17, 128.02, 127.72, 127.54, 126.81, 126.30, 78.90, 76.39, 74.64, 74.60, 74.11, 66.58, 52.16, 52.01, 51.87, 51.78. MS-ESI m/z calcd for C_68_H_60_O_8_ (M)^+^: 1004.43, Found: 1004.55. IR (neat): 822, 949, 1016, 1079, 1174, 1270, 1718, 2930 cm^−1^.

**4**

(4-Benzyloxycarbonylphenyl)-substituted macrocycle **11** (290 mg, 0.29 mmol) was dissolved in 80 mL anhydrous tetrahydrofuran under nitrogen and cooled to −78 ^o^C. The freshly prepared sodium naphthalenide 2.3 mL (2.3 mmol,1.0 M in THF) was added. The reaction was stirred for 2 h at −78 ^o^C, then 2.1 mL I_2_ (1 M solution in THF) was added. After the reaction mixture was warmed up to room temperature, sodium thiosulfate saturated solution was carefully added to remove excess I_2_. 40 mL water was added. After extraction with 3×40 mL dichloromethane, the combined organic phase was washed with 3×40 mL water and dried over sodium sulfate. After removing the solvent under vacuum, the crude yellow solid was used in next step directly without further purification.

To a stirred solution of this 4-benzyloxycarbonylphenyl cycloparaphenylene **12** in a mixture of 50 mL CH_3_OH/ 50 mL THF was added 0.8 g NaOH in 10 mL H_2_O. The reaction mixture was allowed to stir for 18 h at room temperature. 0.1 M HCl was added to the reaction mixture until pH=2. The mixture was then extracted with 3×30 mL dichloromethane, the combined organic phase was washed with 3×30 mL water and dried over anhydrous sodium sulfate. After concentrating in vacuo, the crude yellow solid was purified by column on silica gel using CH_3_OH/DCM=5:95 to give **4** as yellow solid (92 mg, 44% over two steps). ^1^H NMR (400 MHz, CDCl_3_) :δ(ppm) 8.15 (d, *J* = 8.3 Hz, 2H, Ar), 7.93 (d, *J* = 1.9 Hz, 1H, Ar), 7.83 (d, *J* = 8.3 Hz, 2H, Ar), 7.61–7.31 (m, 26H, Ar), 7.16–7.05 (m, 4H, Ar). ^13^C NMR (100 MHz, CDCl_3_): δ(ppm) 170.13, 146.83, 137.73 (multiple overlapping peaks), 134.87, 130.76, 130.22, 129.91, 127.53 (multiple overlapping peaks), 125.33. MALDI-TOF m/z calcd for C_55_H_36_O_2_ (M)^+^: 728.27, Found: 728.3067. IR (neat): 800, 1075, 1260, 1484, 1718, 1772, 2854, 2925 cm^−1^.

**2. Supplementary data of CPPs and their assemblies**


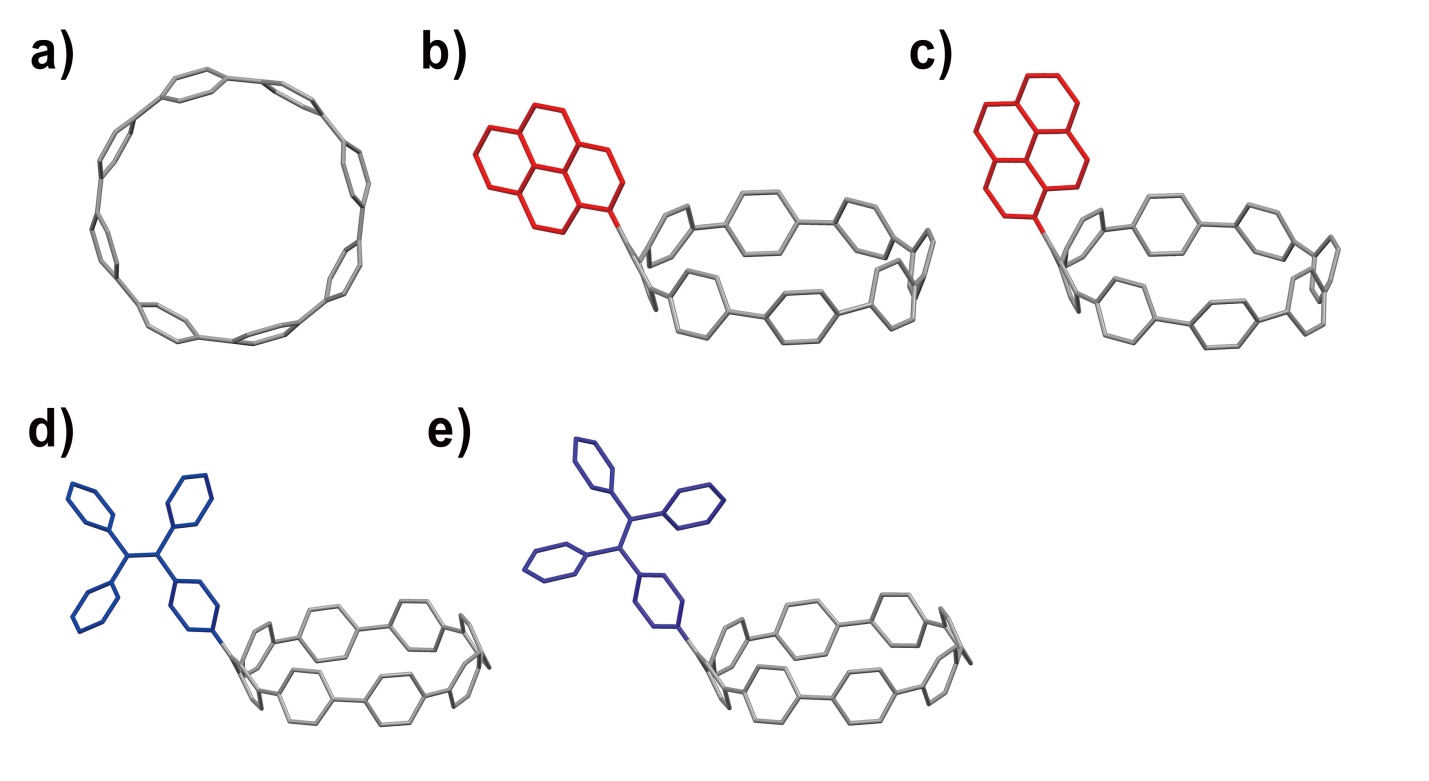


**Fig. S1. Spatial structure of CPPs.** Spatial structure of [8]CPP (a), two isomers of [8]CPP-pyrene (b, c) and two isomers of [8]CPP-TPE (d, e) determined by DFT methods using RB3LYP/6-31G(d).


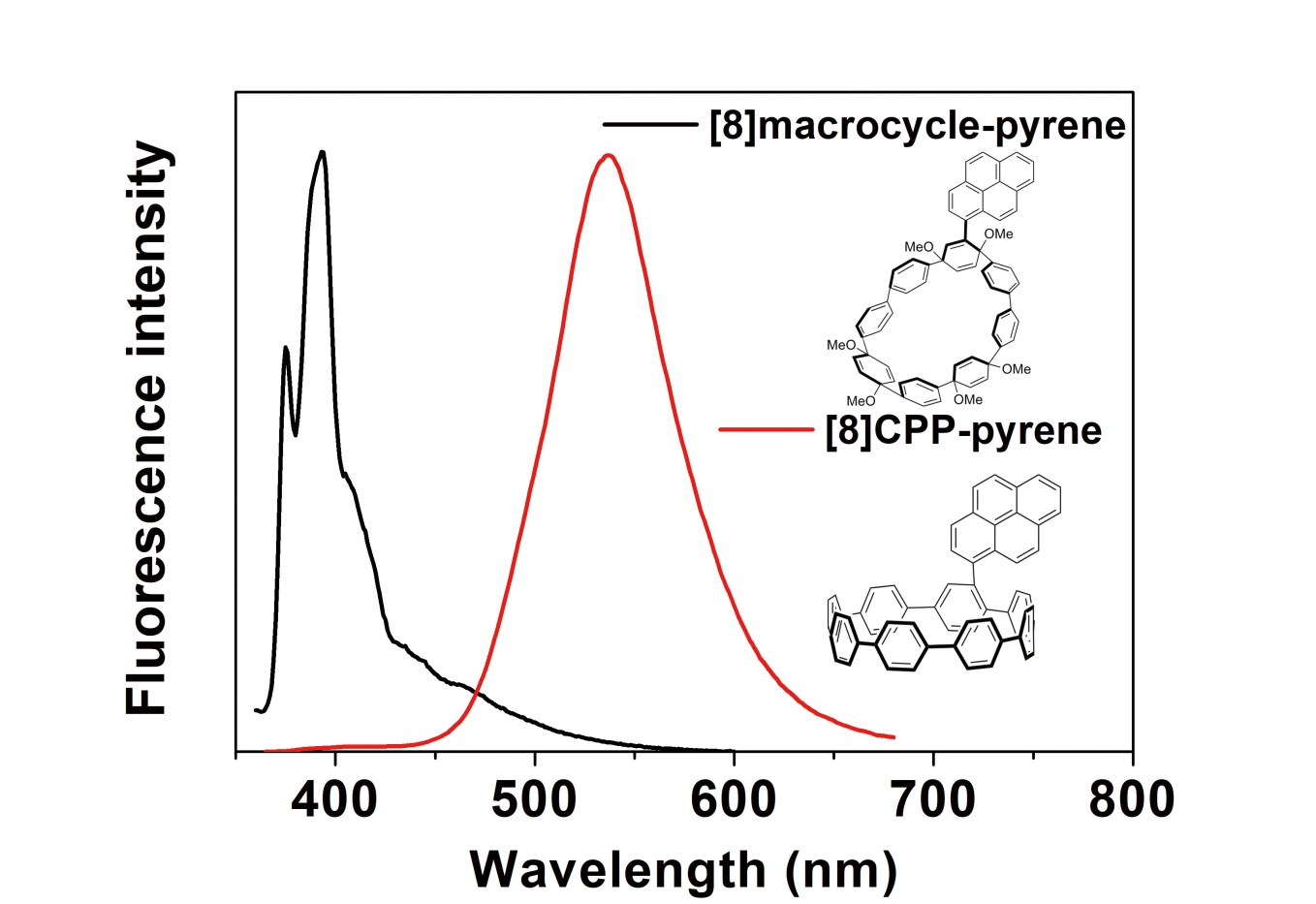


**Fig. S2. Fluorescence spectra of [8]macrocycle-pyrene.** Normalized fluorescence emission spectra of [8]macrocycle-pyrene **8** (λ_ex_ = 350 nm) and [8]CPP-pyrene (λ_ex_ = 340 nm).


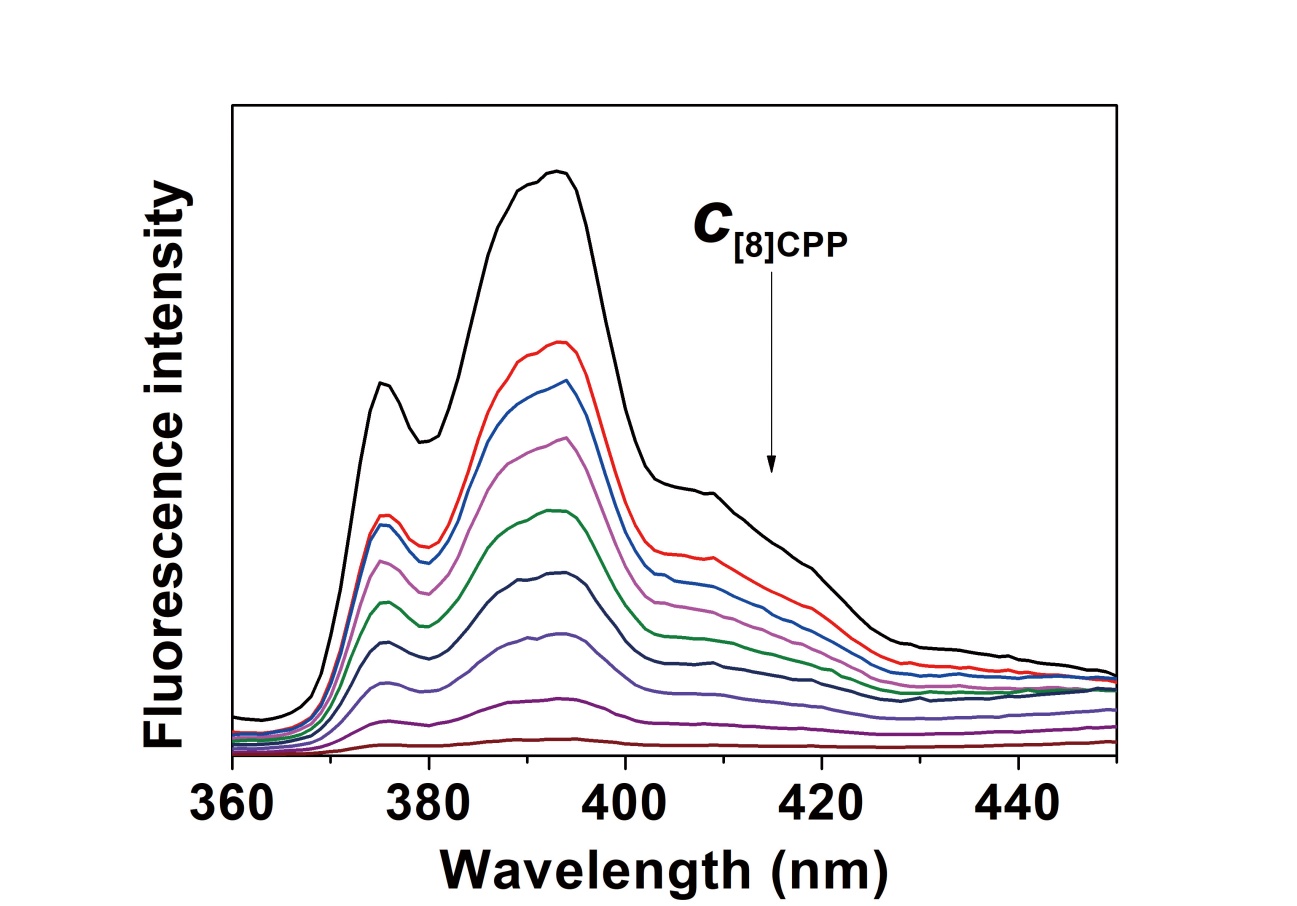


**Fig. S3. Fluorescence quench of pyrene in the presence of [8]CPP.** Fluorescence emission spectra of pyrene (6.2×10^−4^ M, λ_ex_ = 350 nm) in the presence of [8]CPP in THF with different concentrations. The concentrations of [8]CPP are 0.00, 0.06, 0.12, 0.24, 0.30, 0.36, 0.60, 0.90 and 1.50 (×10^−4^ M) from the top to the bottom.


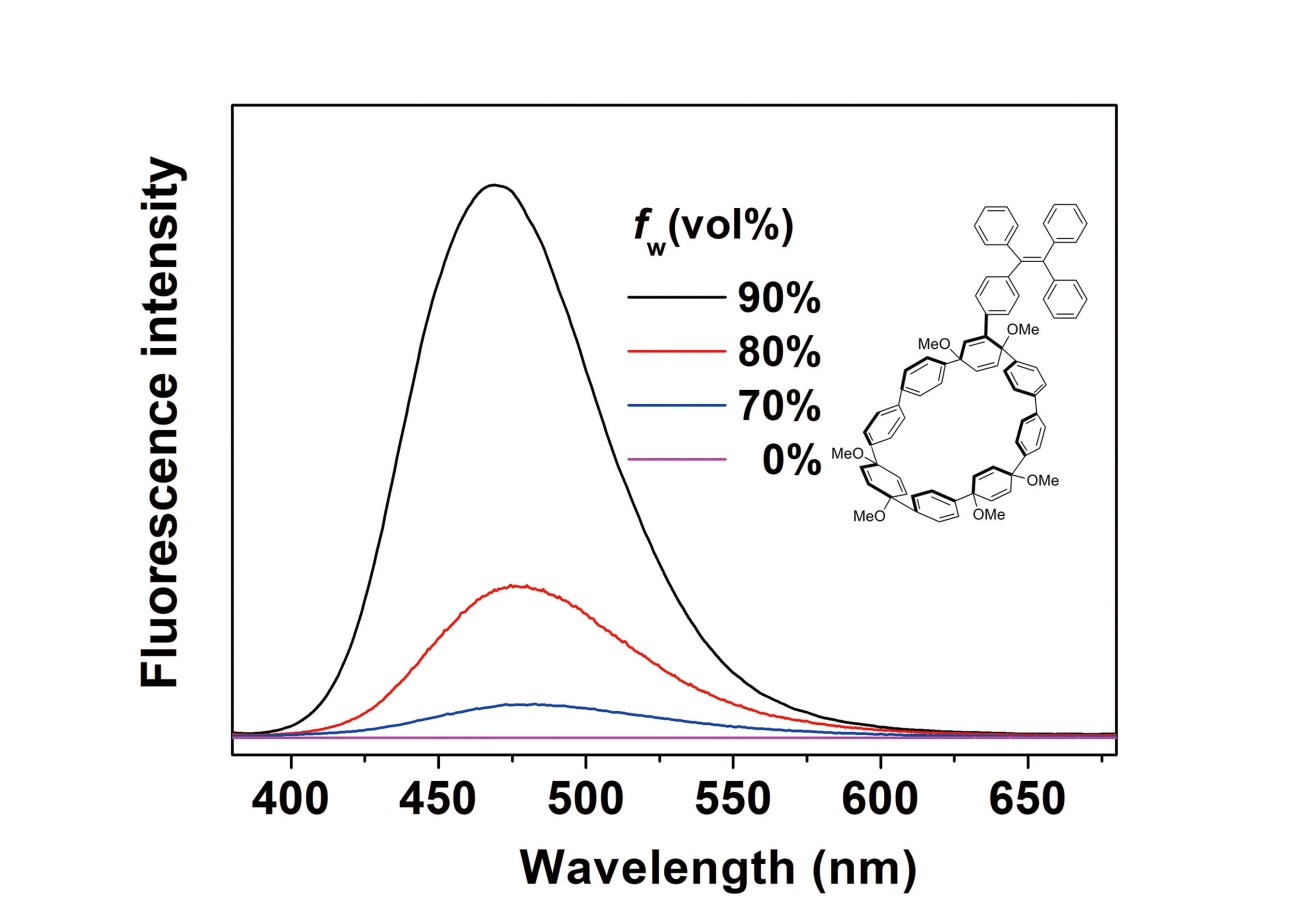


**Fig. S4.** **Aggregation-induced emission of [8]macrocycle-TPE.** Emission spectra of [8]macrocycle-TPE **10** in THF/H_2_O mixed solvent with different fractions of water. [8]macrocycle-TPE concentration: 2×10^−4^ M, λ_ex_ = 360 nm.


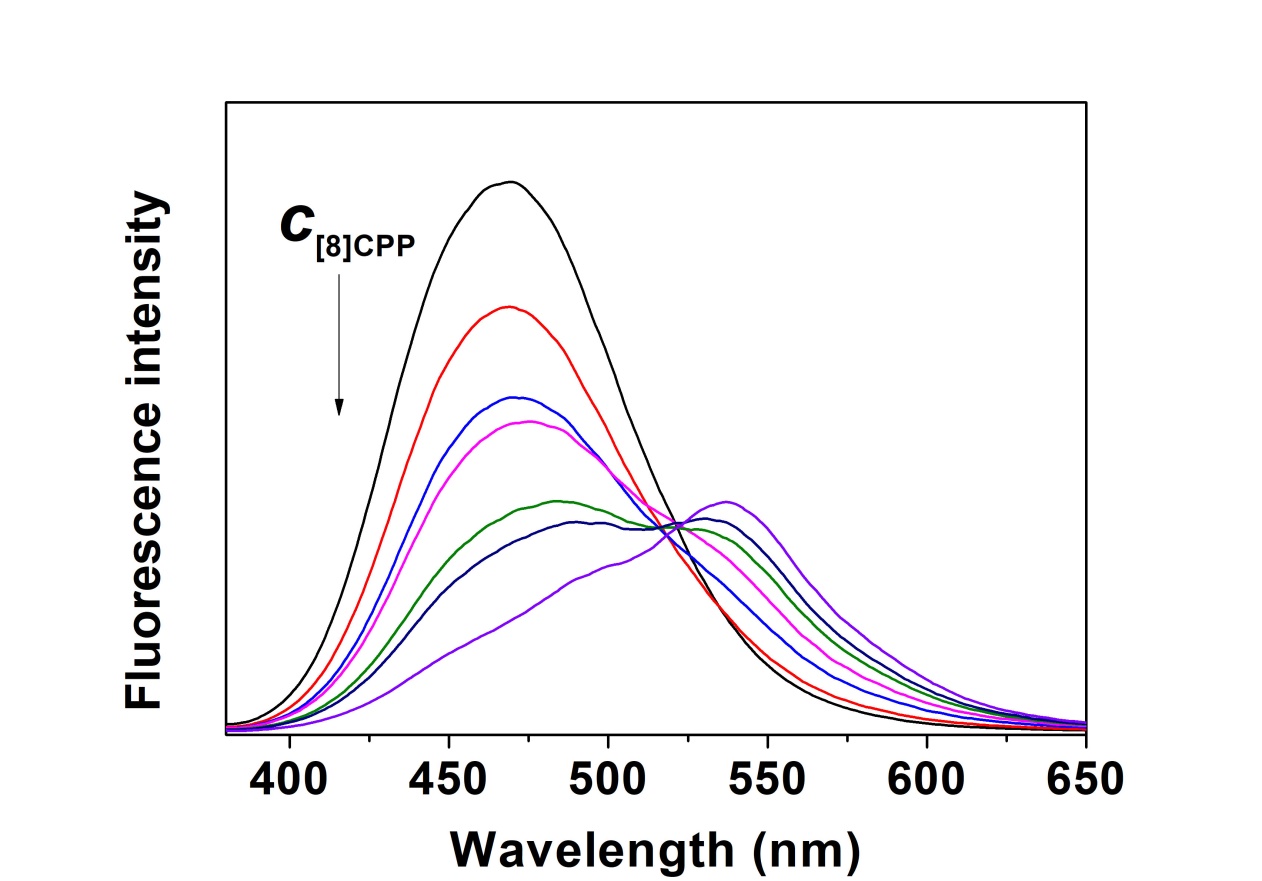


**Fig. S5. Fluorescence quench of TPE in the presence of [8]CPP.** Fluorescence emission spectra of TPE (1.5×10^−3^ M, λ_ex_ = 360 nm) in the presence of [8]CPP in THF/H_2_O = 1/9 mixed solvent with different concentrations. The concentrations of [8]CPP are 0.0, 0.6, 1.2, 1.8, 2.4, 3.0 and 4.2 (×10^−5^ M) from the top to the bottom.


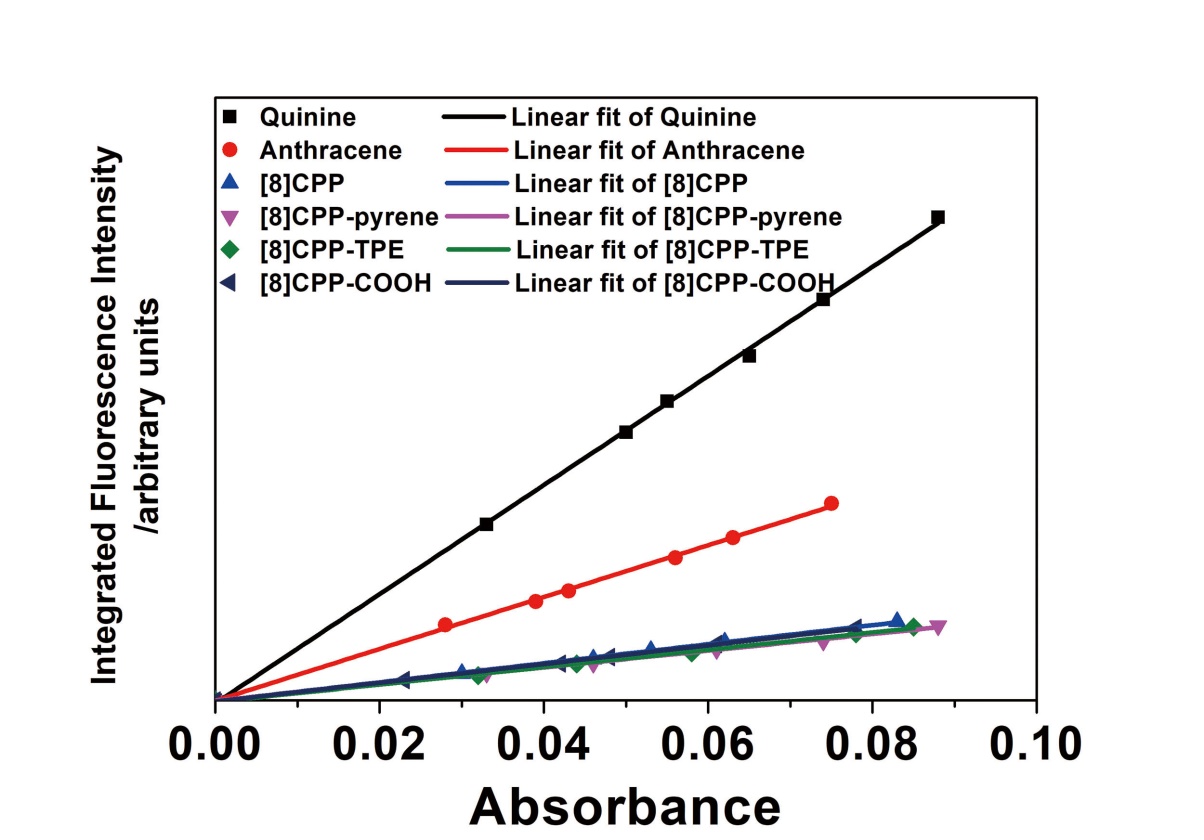


**Fig. S6. Quantum yield of CPPs.** Quantum yield measurement of [8]CPP (Φ = 0.10), [8]CPP-pyrene (Φ = 0.09), [8]CPP-TPE (Φ = 0.09), [8]CPP-COOH (Φ = 0.10).


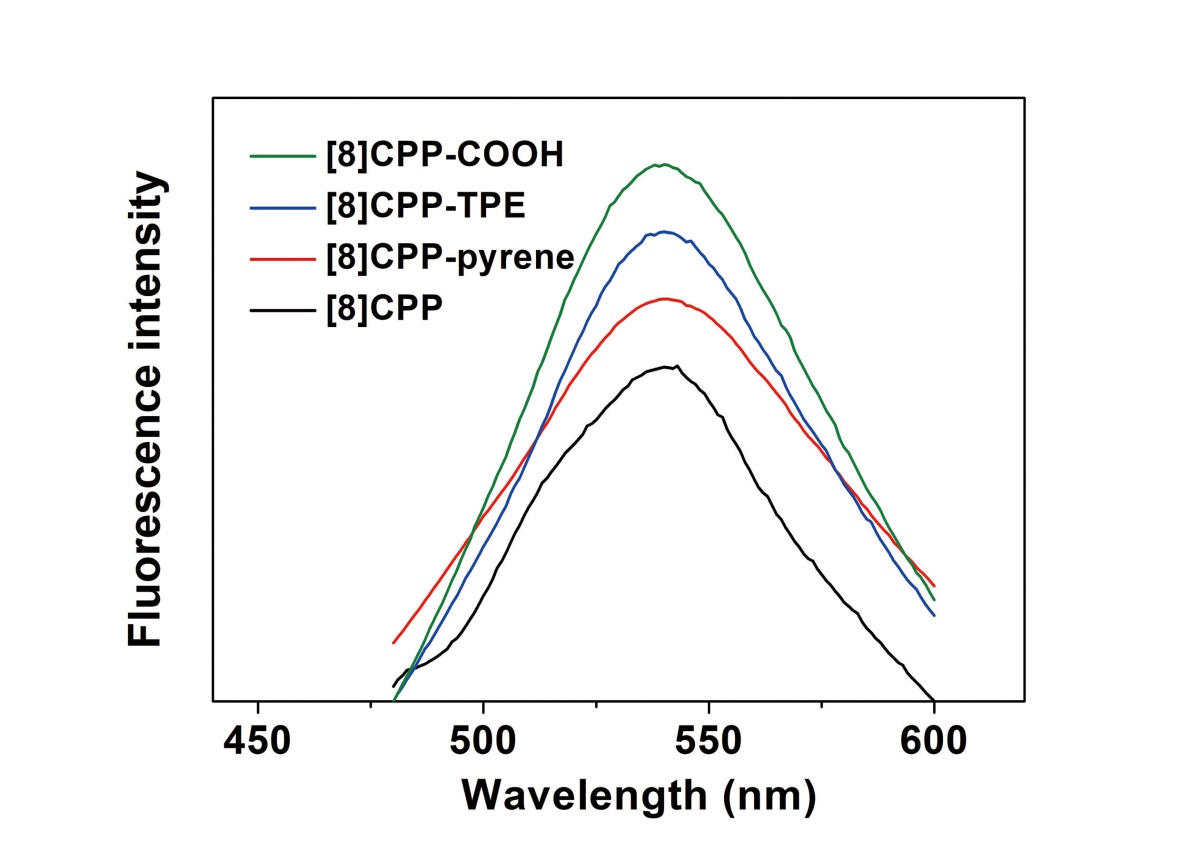


**Fig. S7. Fluorescence emission spectra of CPPs in concentrated THF solution.** Normalized fluorescence emission spectra of [8]CPP (3.6×10^−3^ M), [8]CPP-pyrene (3.5×10^−3^ M), [8]CPP-TPE (3.3×10^−3^ M), [8]CPP-COOH (4.0×10^−3^ M) in concentrated THF solution. At this high concentration, the excitation spectrum of CPP was red shifted to about 455 nm, thus the excitation wavelength was choosen to be λ_ex_ = 455 nm.


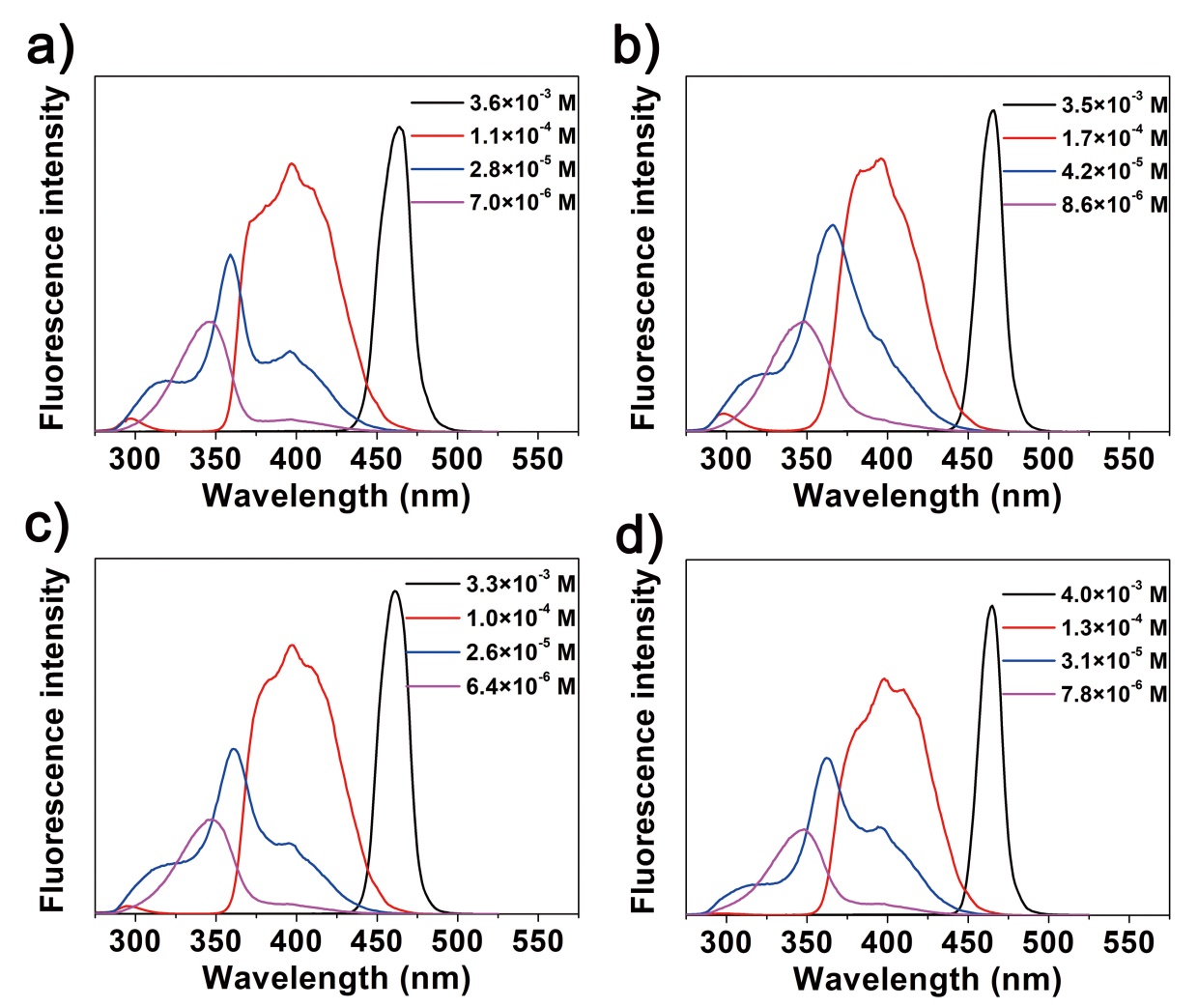


**Fig. S8.** **Fluorescence excitation spectra of CPPs at different concentration.** Fluorescence excitation spectra of [8]CPP (a, λ_em_ = 540 nm), [8]CPP-pyrene (b, λ_em_ = 540 nm), [8]CPP-TPE (c, λ_em_ = 540 nm), [8]CPP-COOH (d, λ_em_ = 540 nm) at different concentration in THF.


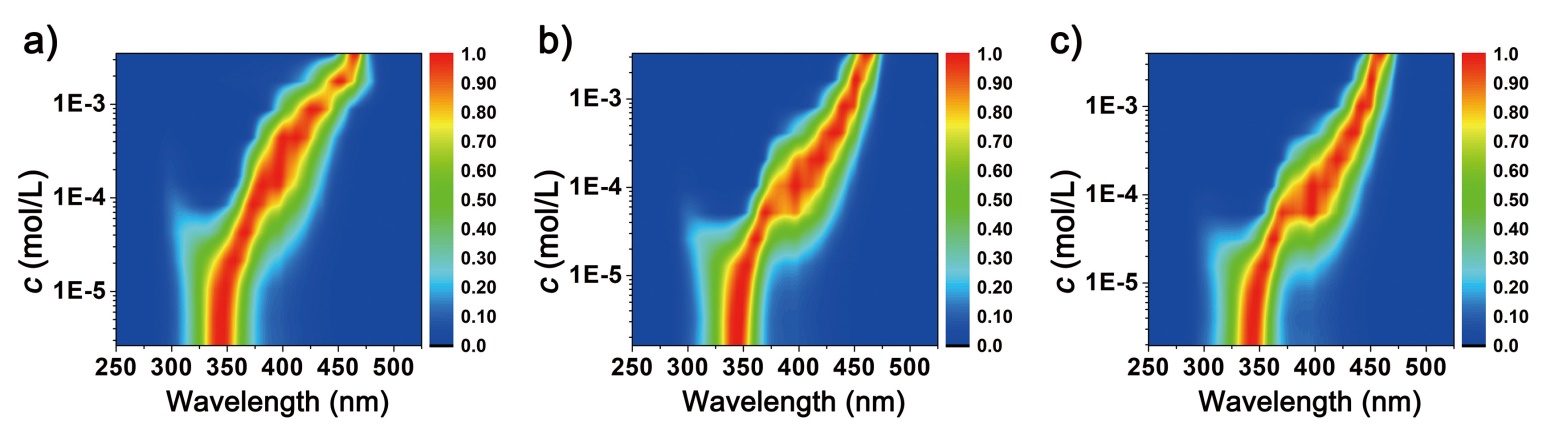


**Fig. S9. Red-shift of CPP excitation spectra in different concentration.** Normalized color plot of the concentration-dependent fluorescence excitation spectra of [8]CPP-pyrene (a, λ_em_ = 540 nm), [8]CPP-TPE (b, λ_em_ = 540 nm), [8]CPP-COOH (c, λ_em_ = 540 nm) between 10^−3^ M to 10^−6^ M in THF.

**Table S1. Major bond lengths, dihedral angles for [8]CPP determined**

**by DFT methods using RB3LYP/6-31G(d)**

| **Parameter** | **[8]CPP** |
| --- | --- |
| Bond length |  |
| C_ipso_-C_ipso_ | 1.4866 Å |
| C_ipso_-C_ortho_ | 1.4074 Å |
| C_ortho_-C_ortho_ | 1.3914 Å |
| Dihedral angel |  |
| C_ortho_–C_ipso_–C_ipso_–C_ortho_ (°) | 30.90, -30.89, 30.90, -30.89, 30.90, -30.90, 30.91, -30.90 |

**Table S2. Major bond lengths, dihedral angles for two isomers of [8]CPP-pyrene**

**determined by DFT methods using RB3LYP/6-31G(d)**

| **Parameter** | **[8]CPP-pyrene isomer 1** | **[8]CPP-pyrene isomer 2** |
| --- | --- | --- |
| Bond length |  |  |
| C_ipso_-C_ipso_ | 1.4866 Å | 1.4866 Å |
| C_ipso_-C_ortho_ | 1.4074 Å | 1.4074 Å |
| C_ortho_-C_ortho_ | 1.3912 Å | 1.3915 Å |
| Dihedral angel |  |  |
| C_ortho_–C_ipso_–C_ipso_–C_ortho_ (°) | 29.87, -30.79, 31.14, -30.73, 30.71, 32.23 (close to pyrene), 35.60 (close to pyrene) | 29.02, -31.05, 31.33, -30.63, 30.79, 32.08 (close to pyrene), 34.42 (close to pyrene) |
| Bond length (pyrene) |  |  |
| C_ipso_-C_ipso_ | 1.4928 Å | 1.4924 Å |
| C_ipso_-C_ortho_ | 1.4226 Å | 1.4219 Å |
| C_ortho_-C_ortho_ | 1.4046 Å | 1.4040 Å |
| C_ortho_-C_pyrene_ | 1.4923 Å | 1.4929 Å |
|  |  |  |
| Dihedral angel |  |  |
| C_ortho_–C_ipso_–C_ipso_–C_ortho_ (°) | 38.12 | 37.29 |
| C_ortho_–C_ipso_–C_pyrene_–C_pyrene_ (°) | 53.40 | 54.84 |

**Table S3. Major bond lengths, dihedral angles for two isomers of [8]CPP-TPE**

**determined by DFT methods using RB3LYP/6-31G(d)**

| **Parameter** | **[8]CPP-TPE isomer 1** | **[8]CPP-TPE isomer 2** |
| --- | --- | --- |
| Bond length |  |  |
| C_ipso_-C_ipso_ | 1.4870 Å | 1.4870 Å |
| C_ipso_-C_ortho_ | 1.4079 Å | 1.4079 Å |
| C_ortho_-C_ortho_ | 1.3906 Å | 1.3905 Å |
| Dihedral angel |  |  |
| C_ortho_–C_ipso_–C_ipso_–C_ortho_ (°) | 18.82, 17.19, -34.64, 17.55, 17.50, -34.06 (close to TPE), -36.41 (close to TPE) | 17.21, 17.30, -34.48, 17.24, 19.01, -34.50 (close to TPE), -36.21 (close to TPE) |
| Bond length (TPE) |  |  |
| C_ipso_-C_ipso_ | 1.4936 Å | 1.4934 Å |
| C_ipso_-C_ortho_ | 1.4228 Å | 1.4229 Å |
| C_ortho_-C_ortho_ | 1.4037 Å | 1.4040 Å |
| C_ortho_-C_TPE_ | 1.4867 Å | 1.4868 Å |
|  |  |  |
| Dihedral angel |  |  |
| C_ortho_–C_ipso_–C_ipso_–C_ortho_ (°) | 36.85 | 37.27 |
| C_ortho_–C_ipso_–C_TPE_–C_TPE_ (°) | 41.69 | 40.43 |

**Table S4. Crystal information of [8]CPP nanosheet powders**

**freeze-dried from THF**

| h | k | l | d (cald) / Å | d (obsd)/ Å | *q* (obsd)/nm^-1^ |
| --- | --- | --- | --- | --- | --- |
| 1 | 1 | 1 | 6.07 | 5.97 | 10.52 |
| 2 | 1 | 0 | 4.92 | 4.91 | 12.79 |
| 2 | 0 | 2 | 4.65 | 4.27 | 14.71 |
| 2 | 2 | 0 | 3.37 | 3.51 | 17.89 |
| 2 | 2 | 2 | 3.04 | 3.06 | 20.54 |
| 3 | 2 | 0 | 2.89 | 2.87 | 21.87 |
| 3 | 2 | 1 | 2.78 | 2.68 | 23.44 |
| 3 | 3 | 0 | 2.25 | 2.07 | 30.36 |
| 3 | 4 | 1 | 1.78 | 1.87 | 33.52 |

The cell parameter was calculated by the following formula：

$\frac{1}{d^{2}}=\frac{1}{\sin^{2} \beta}(\frac{h^{2}}{a^{2}}+\frac{k^{2}\sin^{2} \beta}{b^{2}}+\frac{l^{2}}{c^{2}}-\frac{2hl\cos\beta}{ac})$,

where a = 12.93 Å, b=8.01 Å, c=19.36 Å, and β = 105.363

**3. Spectrum data of synthesized intermediates and resulting products**

**3.1 MALDI-TOF spectra**


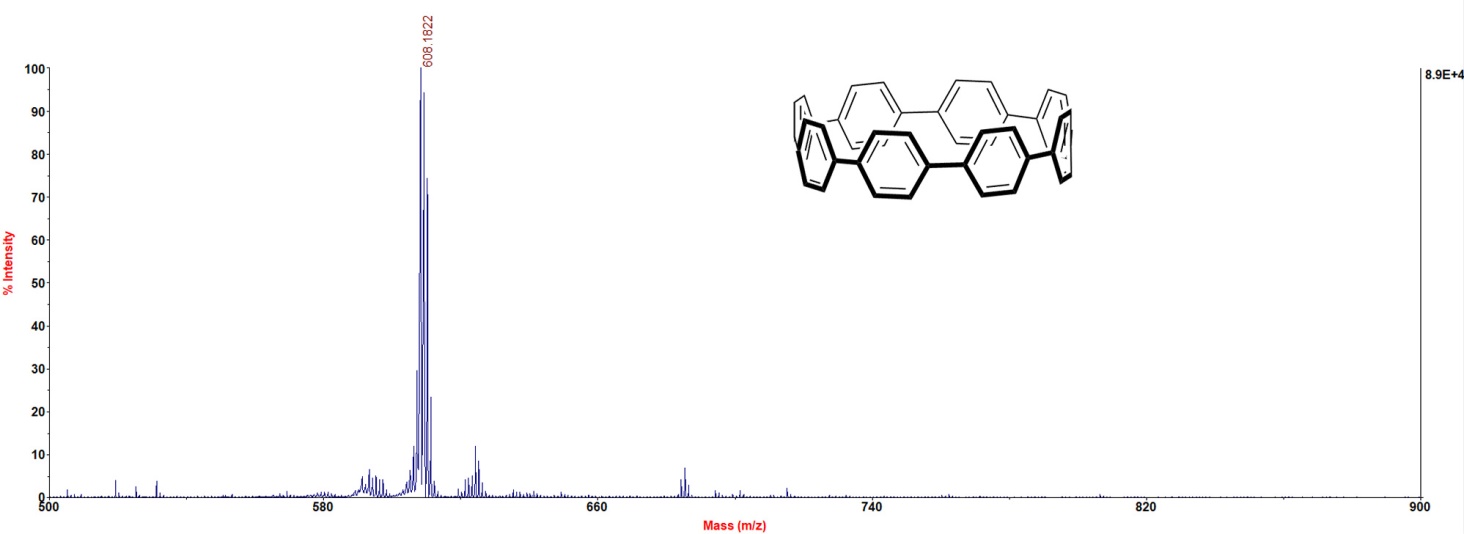


**Fig. S10.** MALDI-TOF spectrum of [8]CPP **1**.


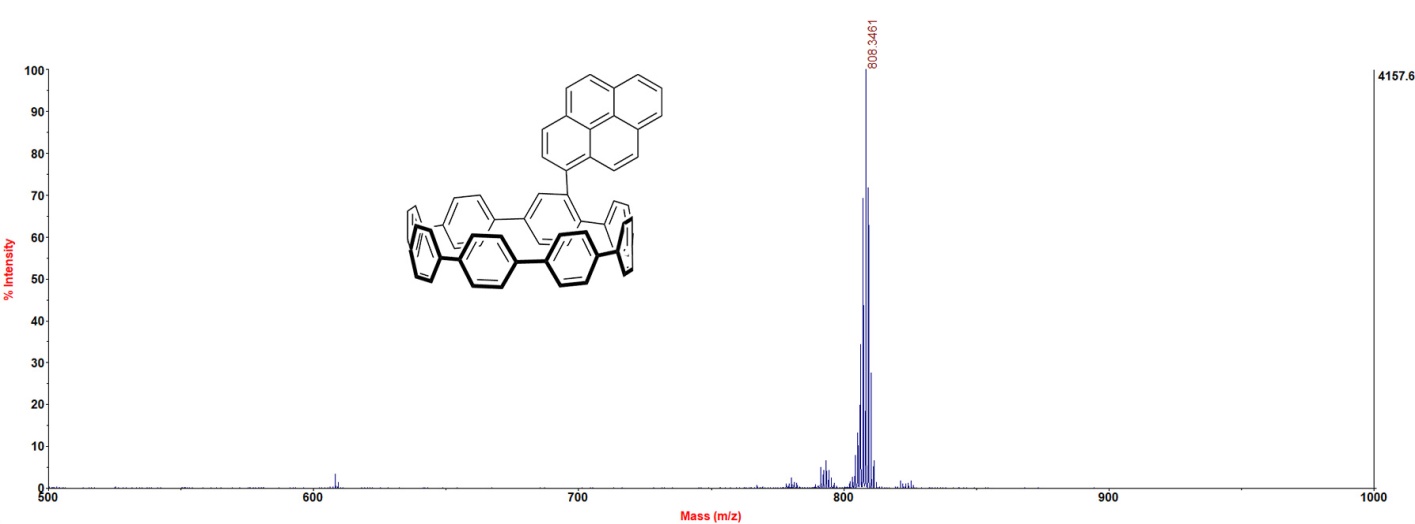


**Fig. S11.** MALDI-TOF spectrum of [8]CPP-pyrene **2**.


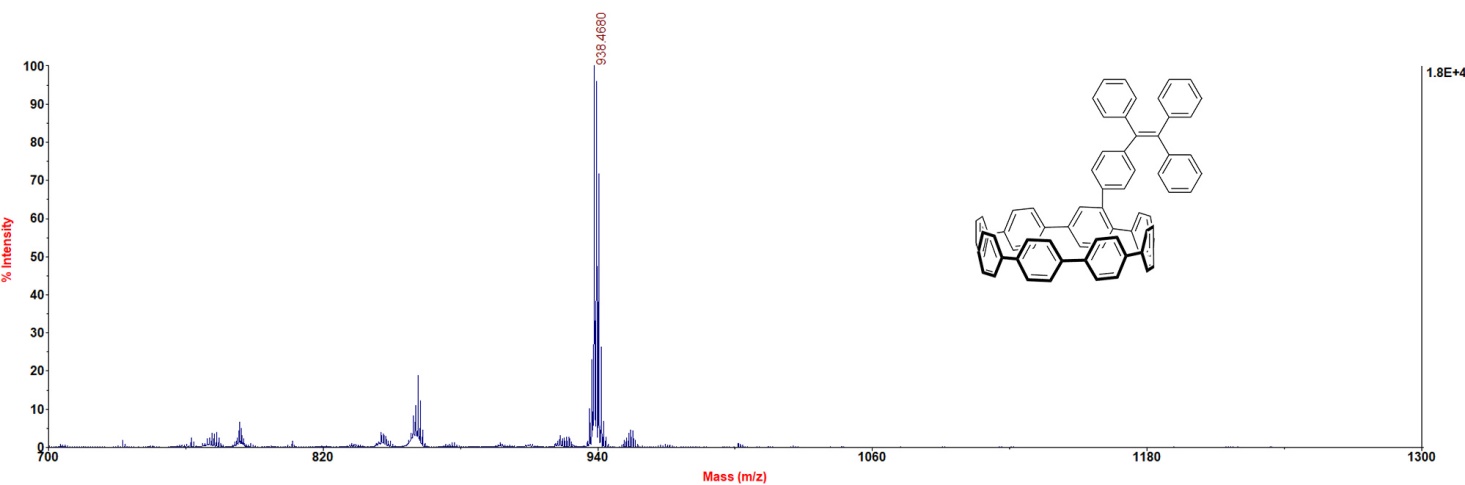


**Fig. S12.** MALDI-TOF spectrum of [8]CPP-TPE **3**.


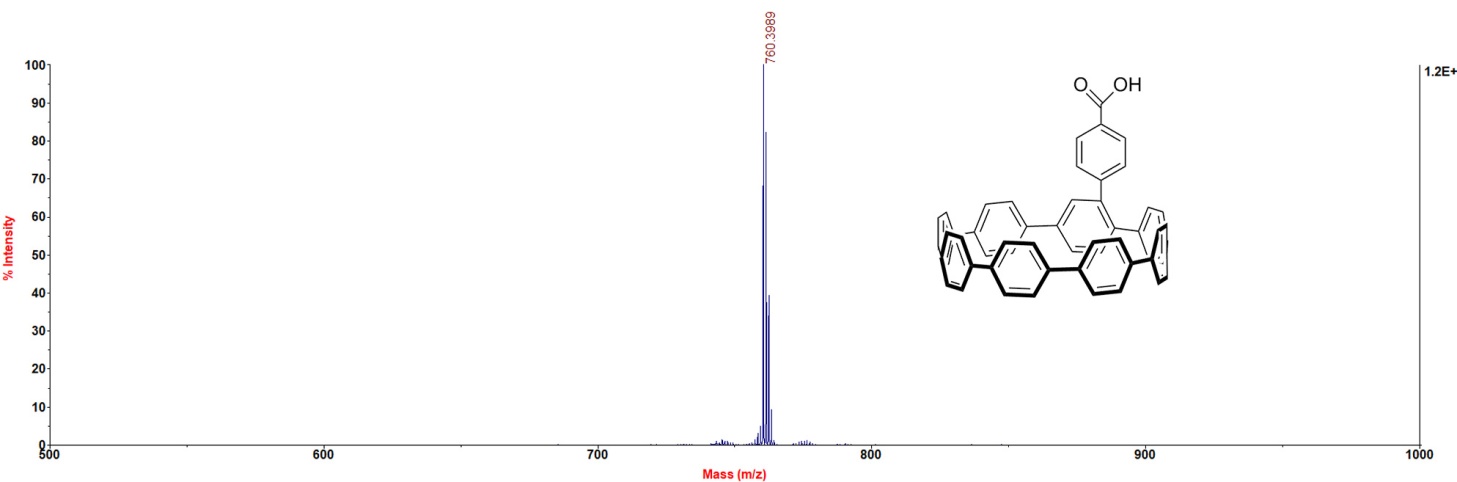


**Fig. S13.** MALDI-TOF spectrum of [8]CPP-COOH **4**.

**3.2 ^1^H NMR and ^13^C NMR spectra**


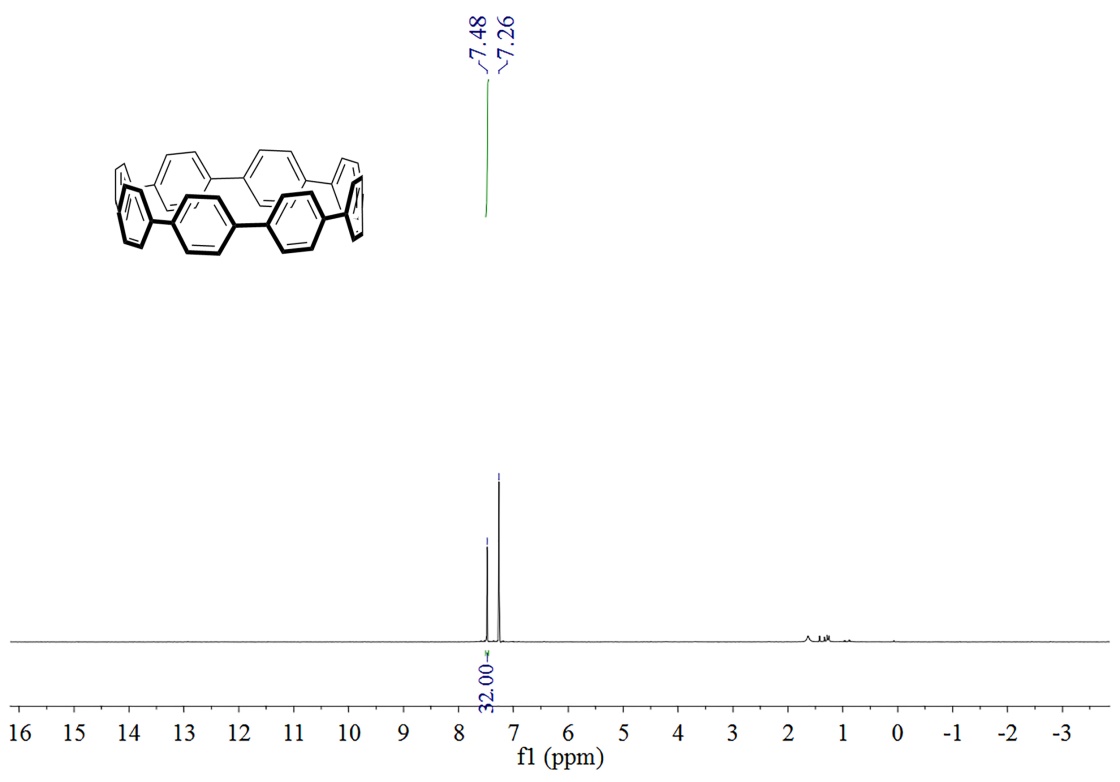


**Fig. S14.** ^1^H NMR spectrum of [8]CPP **1**.


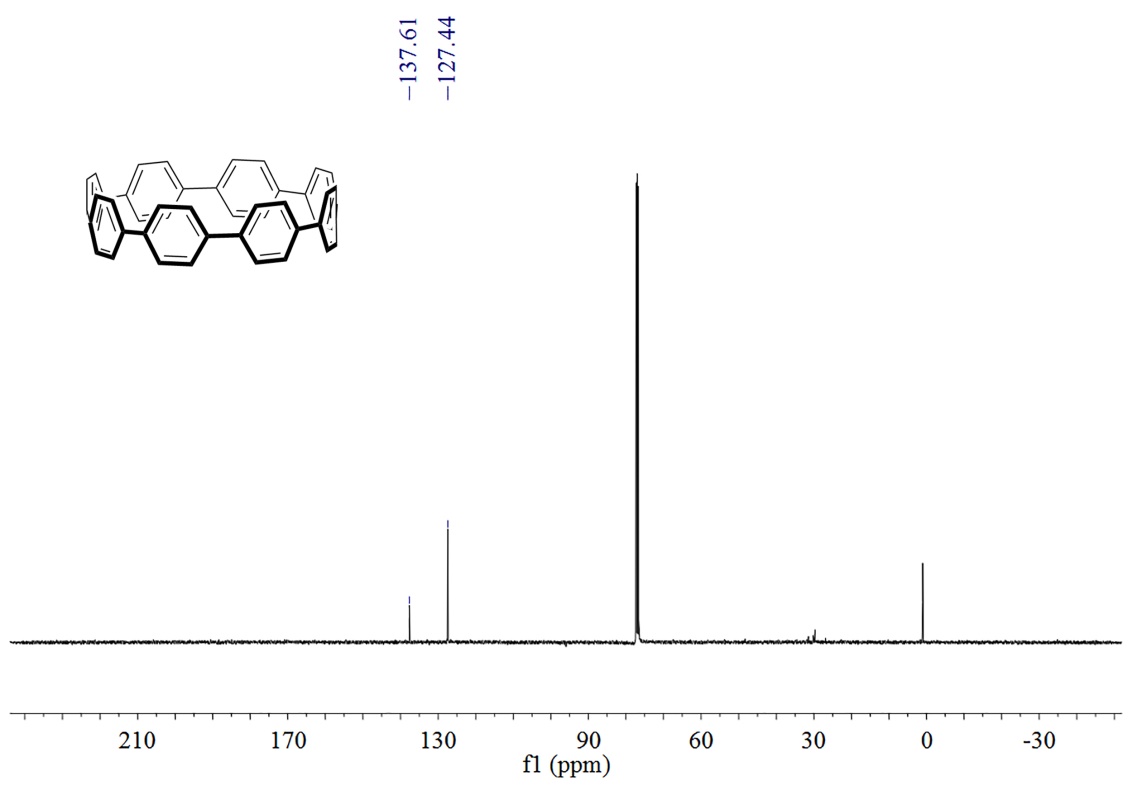


**Fig. S15.** ^13^C NMR spectrum of [8]CPP **1**.


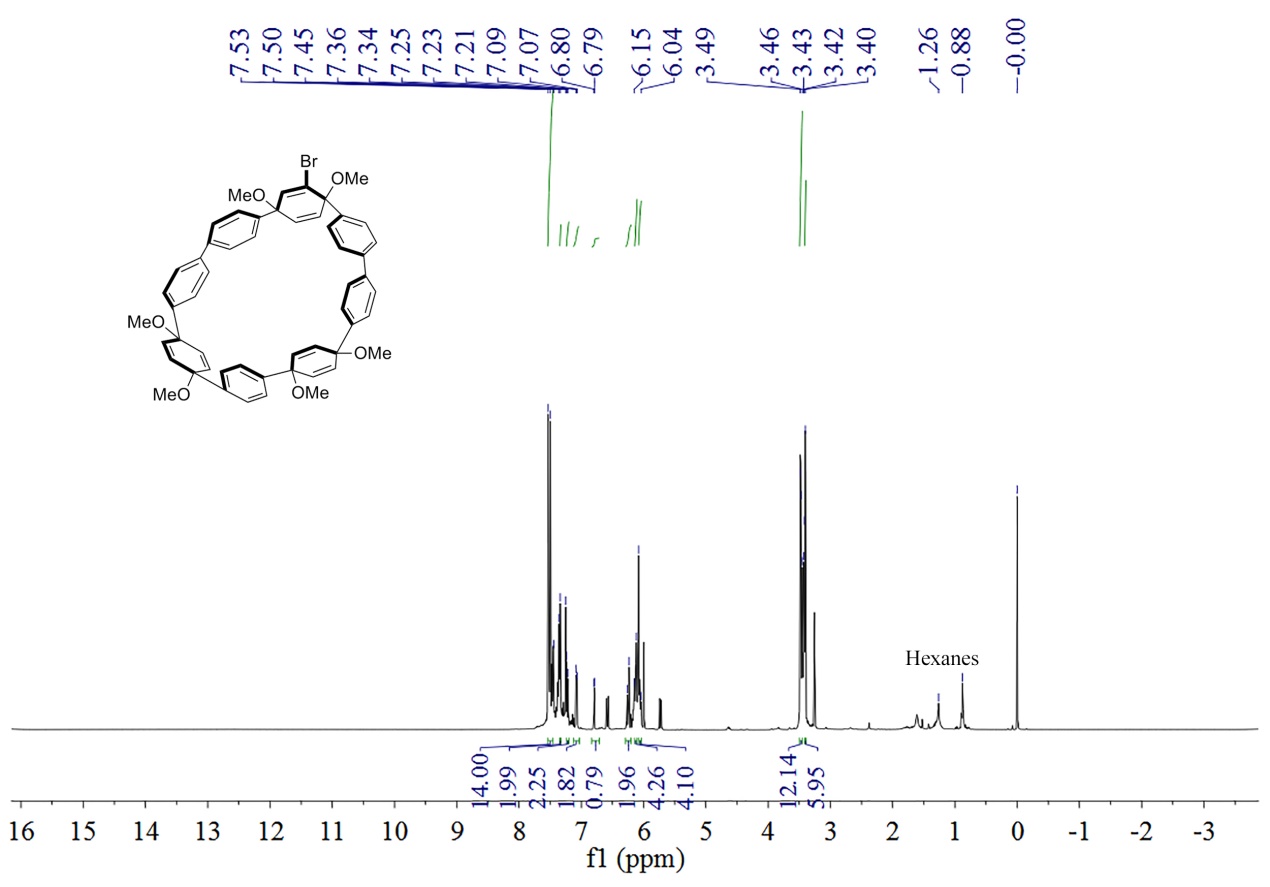


**Fig. S16.** ^1^H NMR spectrum of bromo-substituted macrocycle **5**.


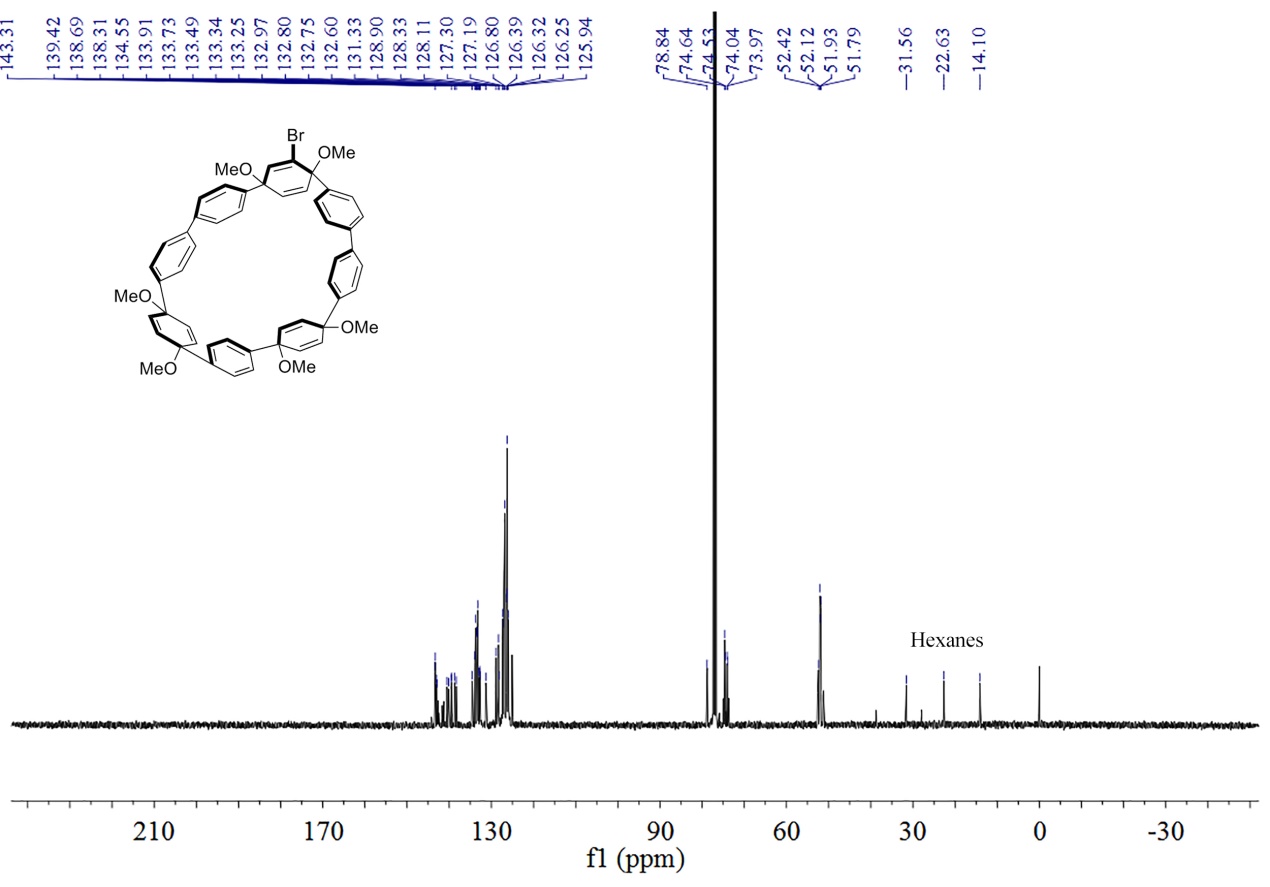


**Fig. S17.** ^13^C NMR spectrum of bromo-substituted macrocycle **5**.


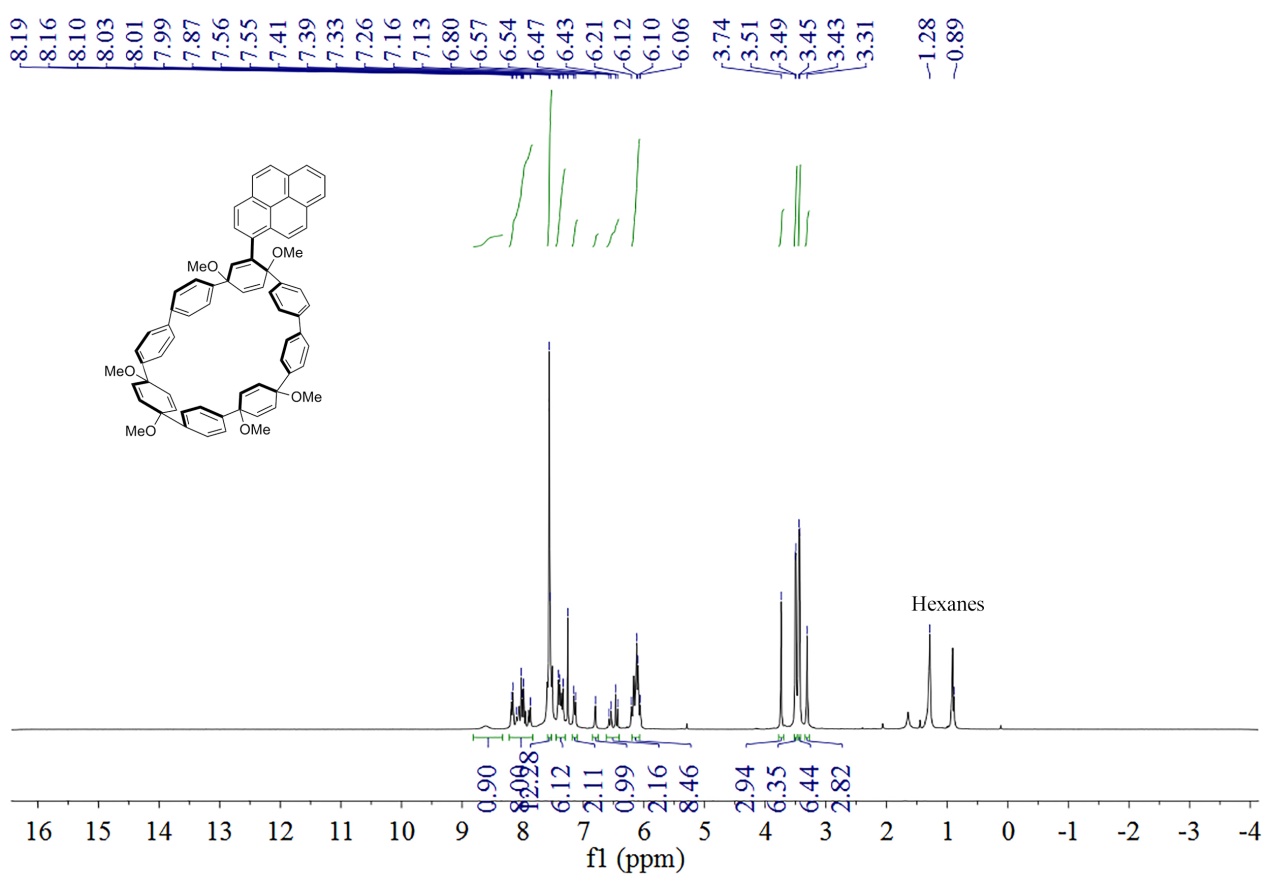


**Fig. S18.** ^1^H NMR spectrum of pyrene-substituted macrocycle **7**.


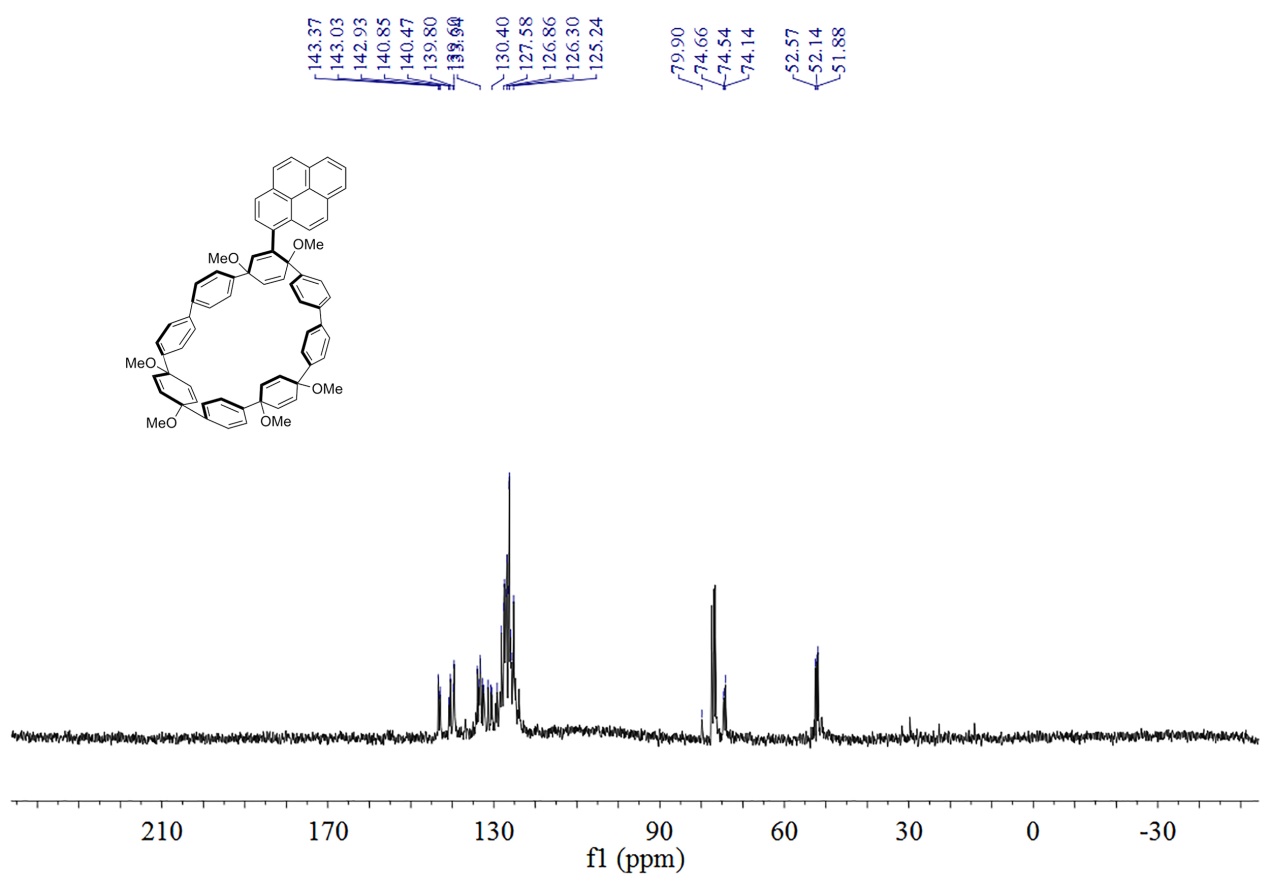


**Fig. S19.** ^13^C NMR spectrum of pyrene-substituted macrocycle **7**.


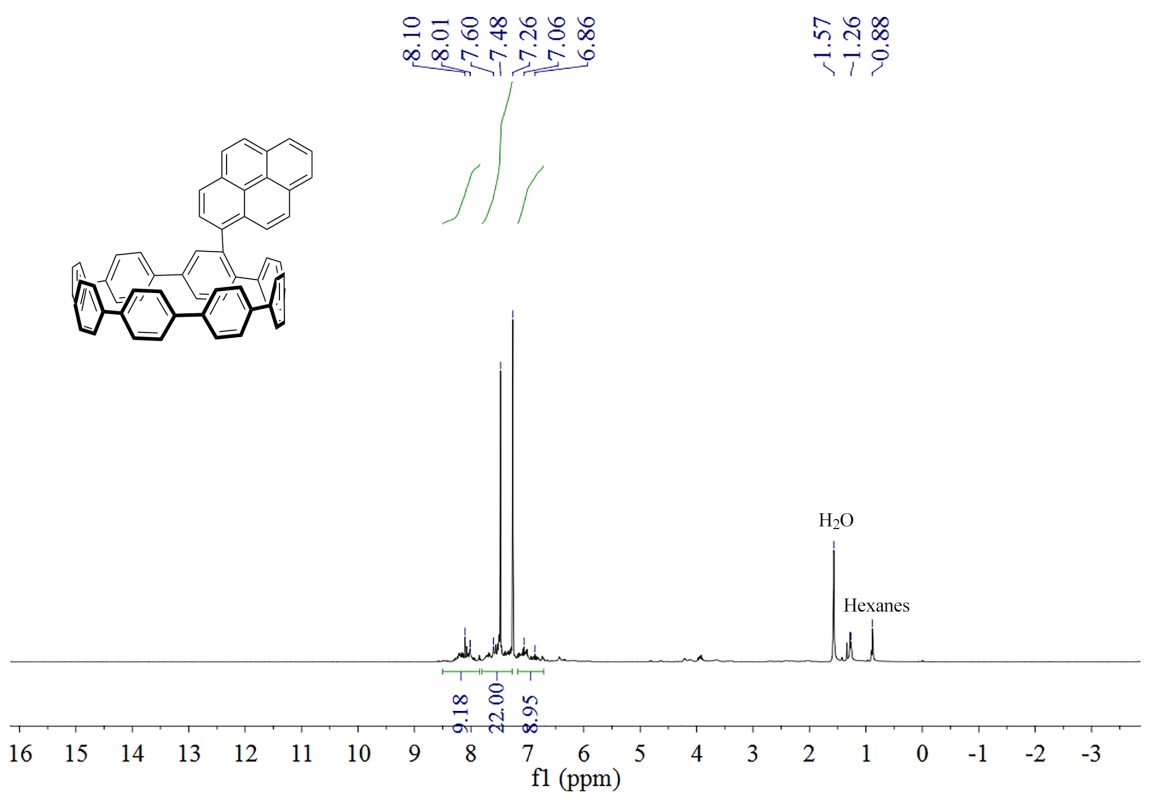


**Fig. S20.** ^1^H NMR spectrum of [8]CPP-pyrene **2**.


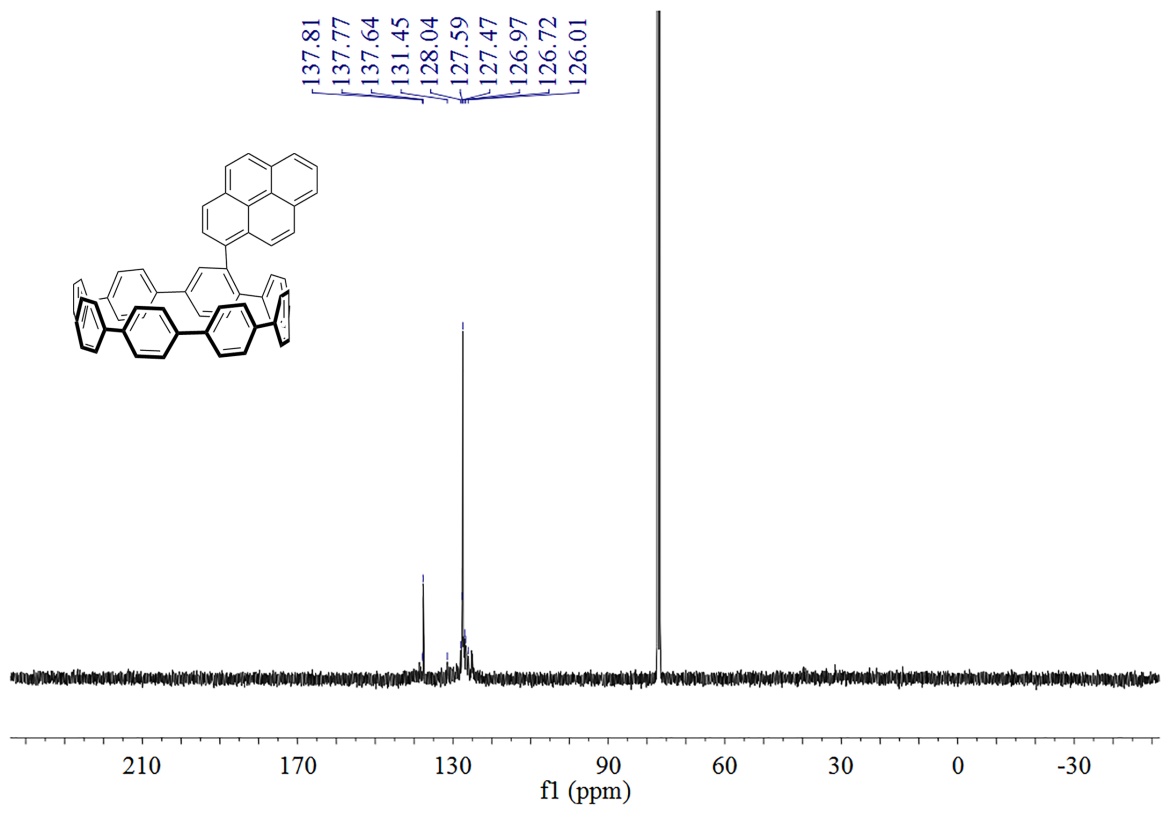


**Fig. S21.** ^13^C NMR spectrum of [8]CPP-pyrene **2**.


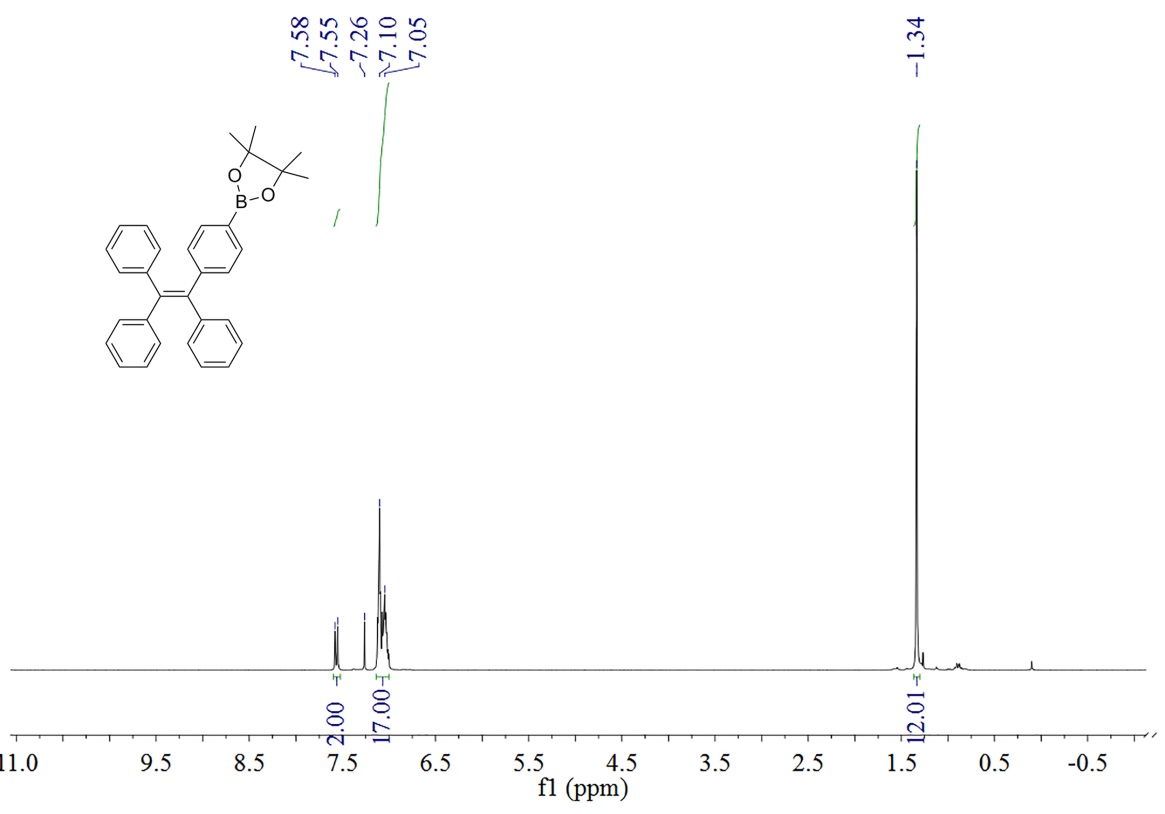


**Fig. S22.** ^1^H NMR spectrum of TPE-Bpin **8**.


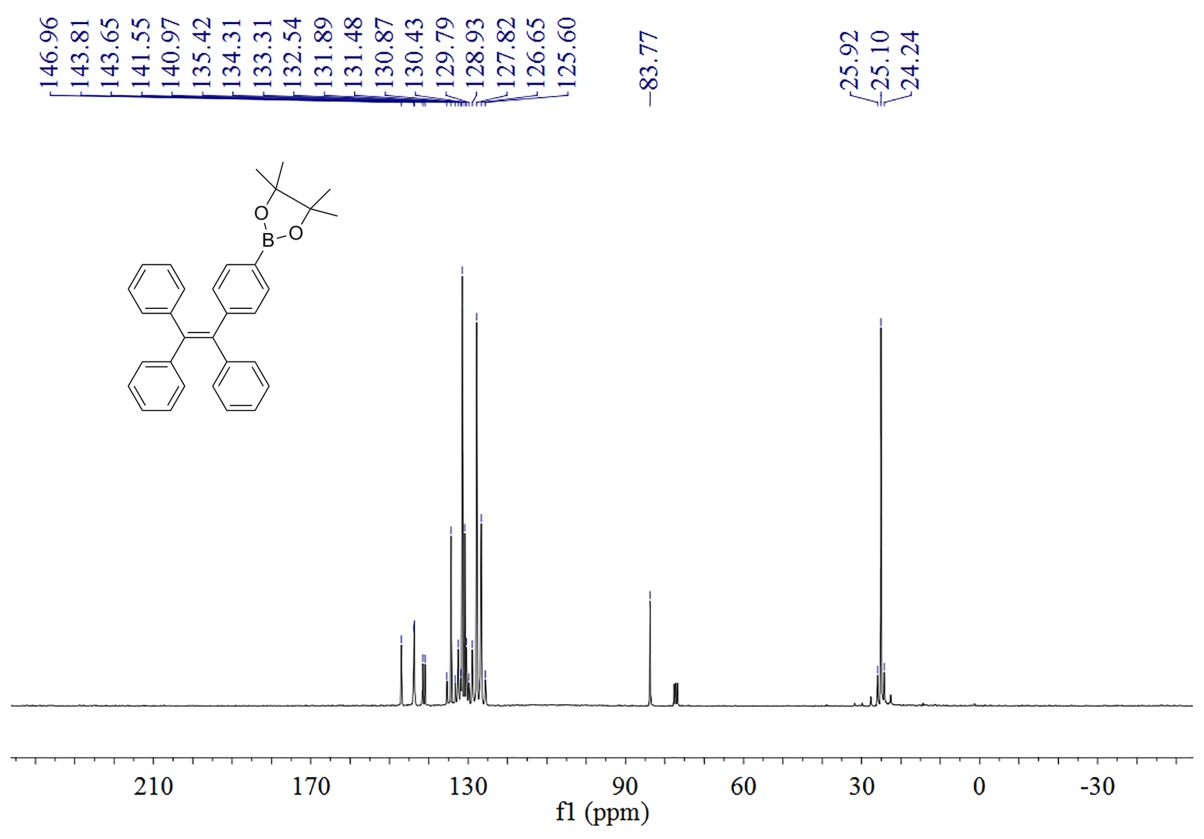


**Fig. S23.** ^13^C NMR spectrum of TPE-Bpin **8**.


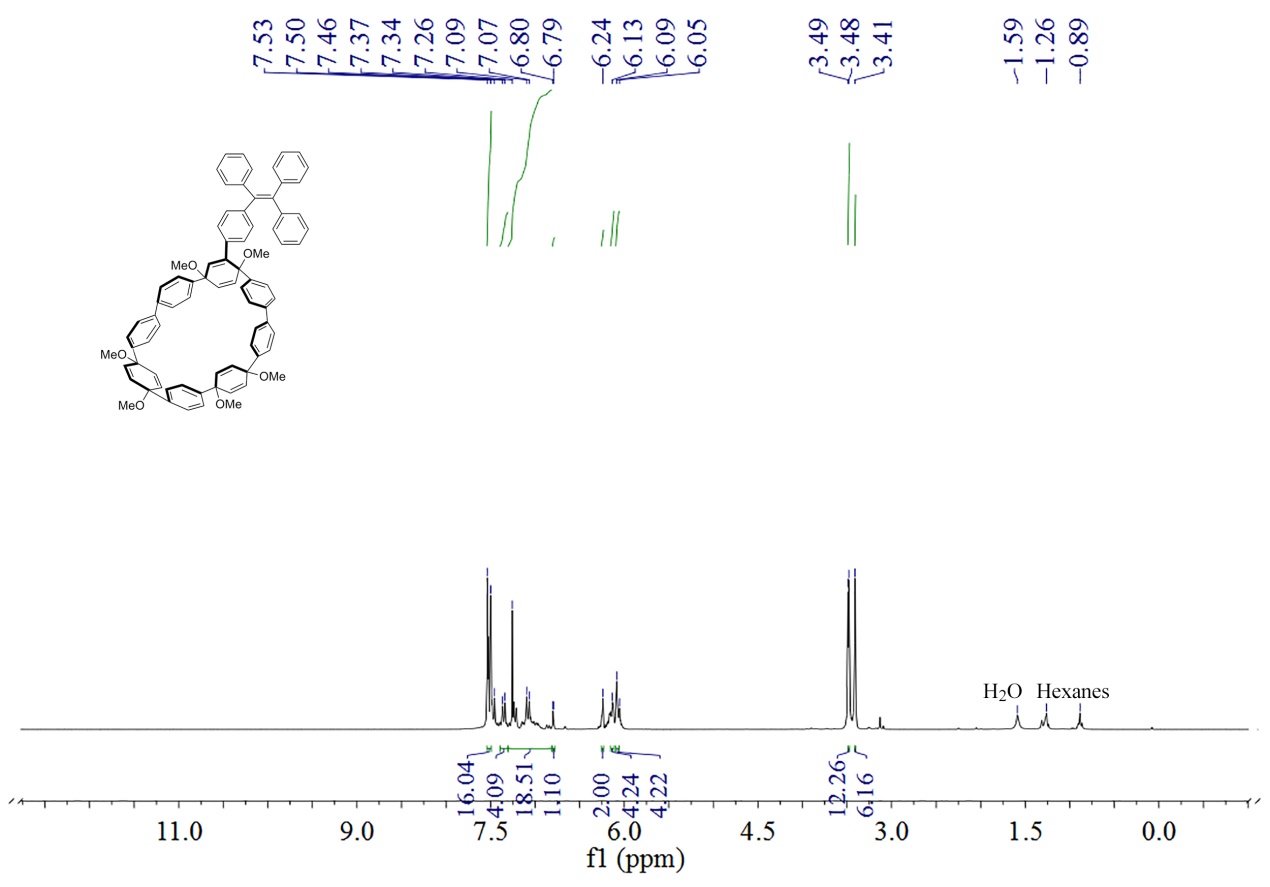


**Fig. S24.** ^1^H NMR spectrum of TPE-substituted macrocycle **9**.


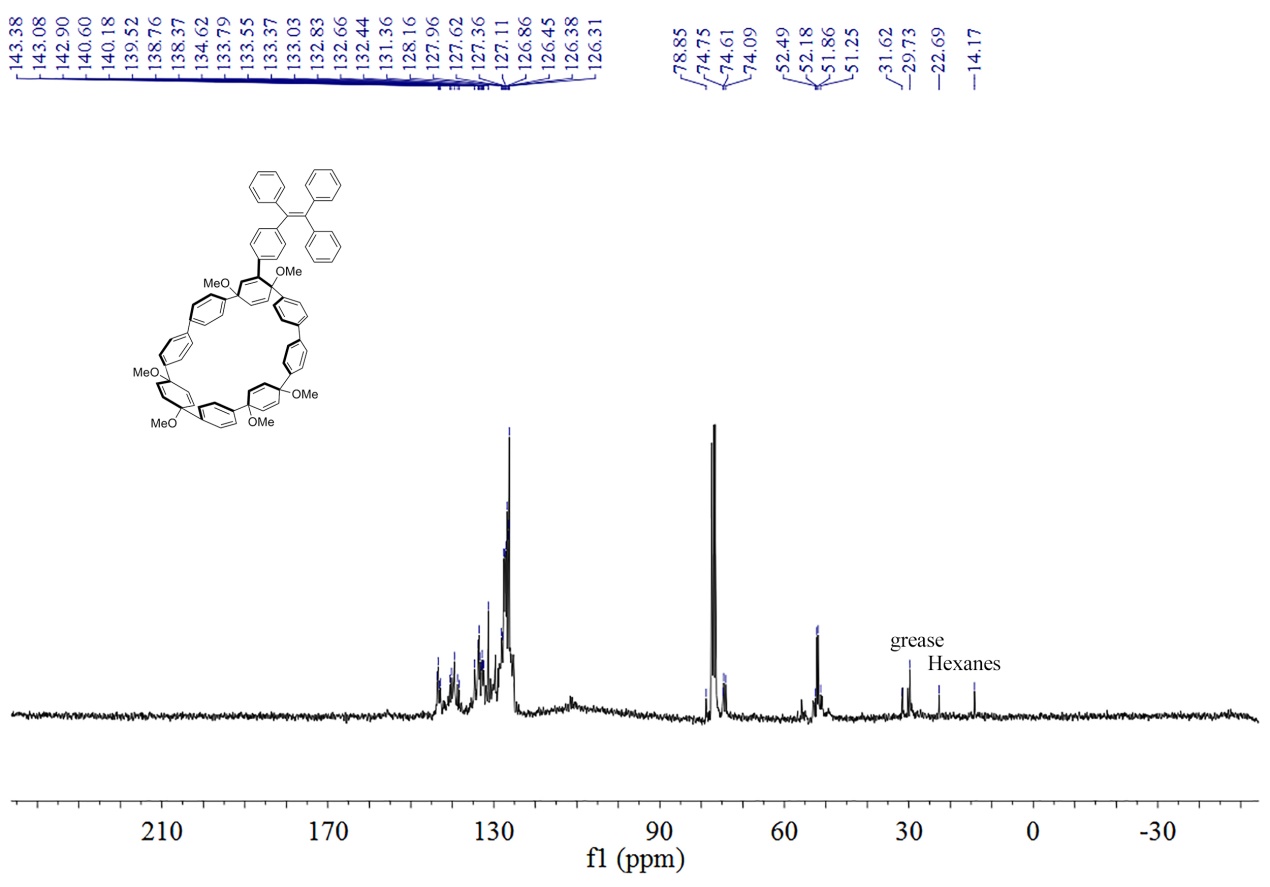


**Fig. S25.** ^13^C NMR spectrum of TPE-substituted macrocycle **9**.


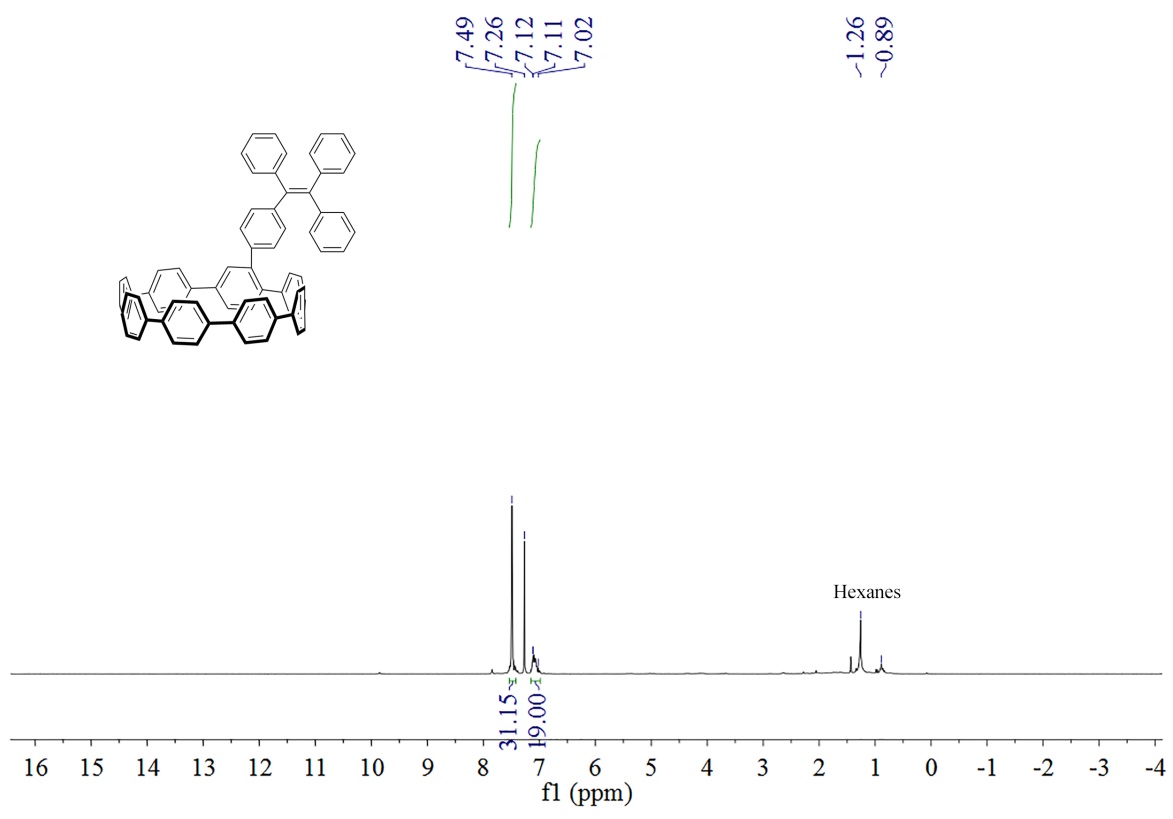


**Fig. S26.** ^1^H NMR spectrum of [8]CPP-TPE **3**.


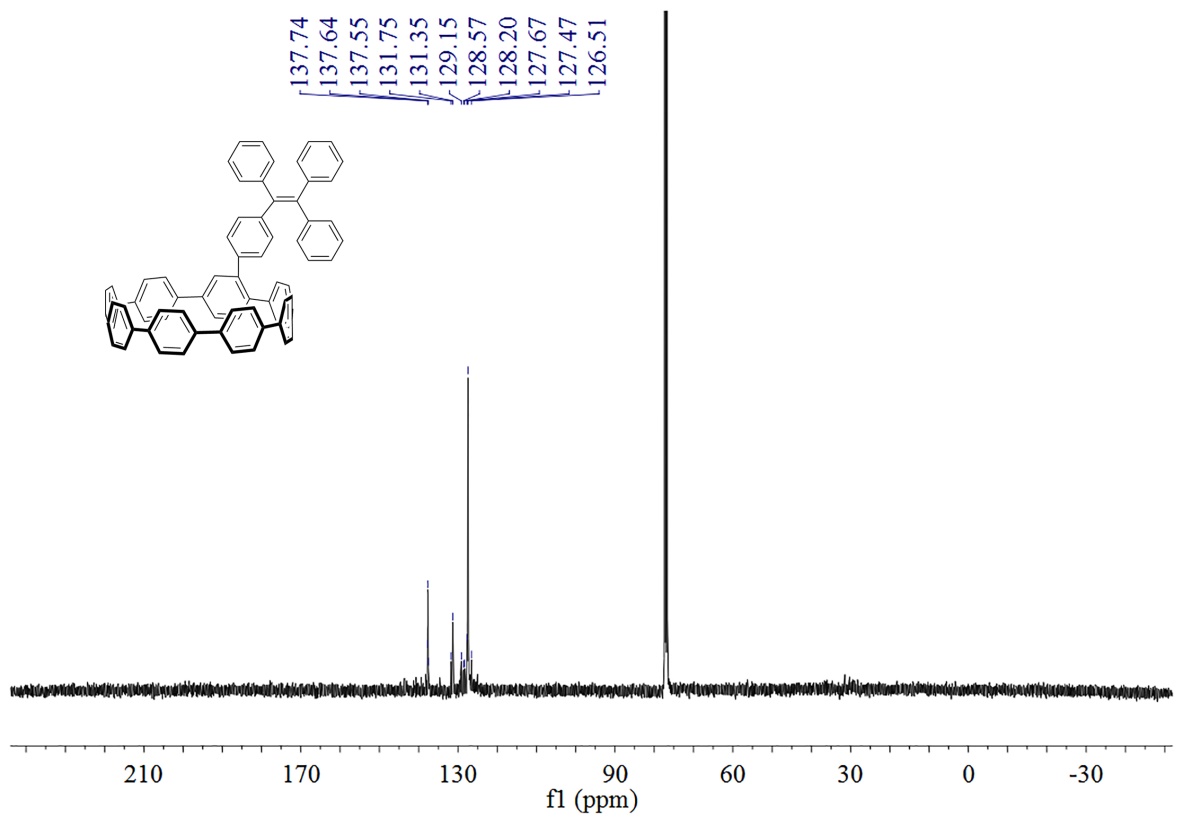


**Fig. S27.** ^13^C NMR spectrum of [8]CPP-TPE **3**.


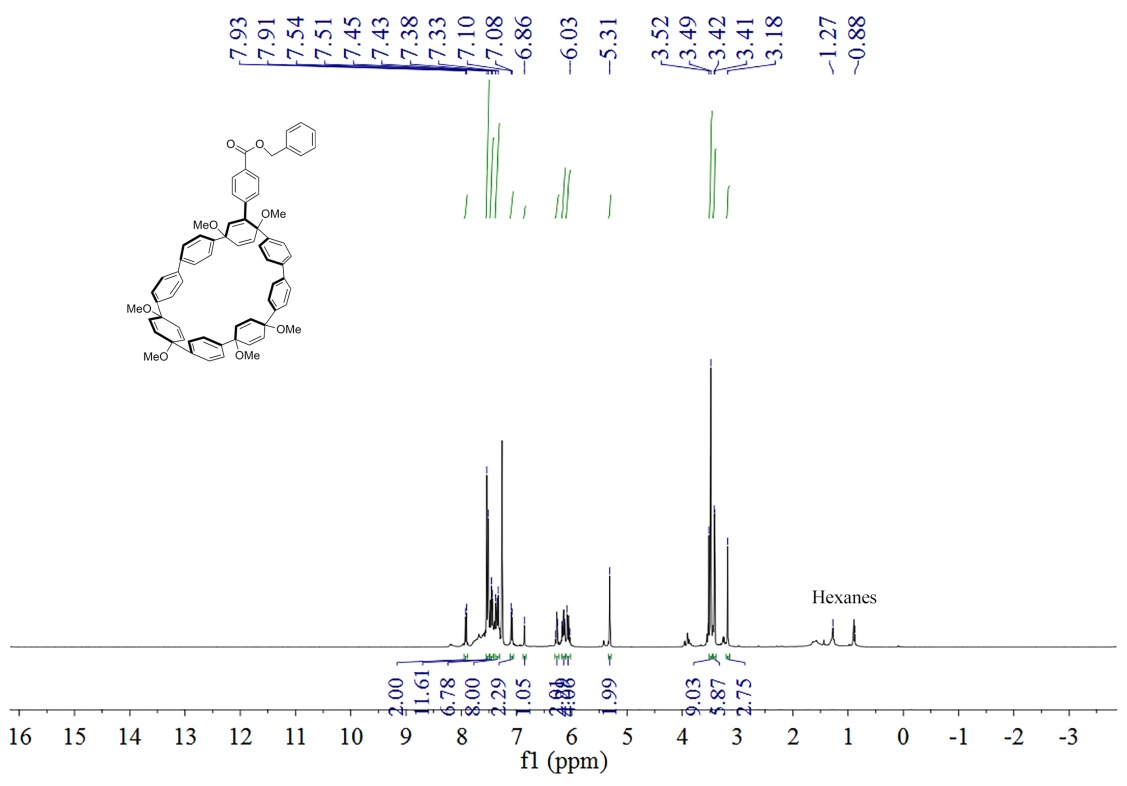


**Fig. S28.** ^1^H NMR spectrum of (4-Benzyloxycarbonylphenyl)- substituted macrocycle **11**.


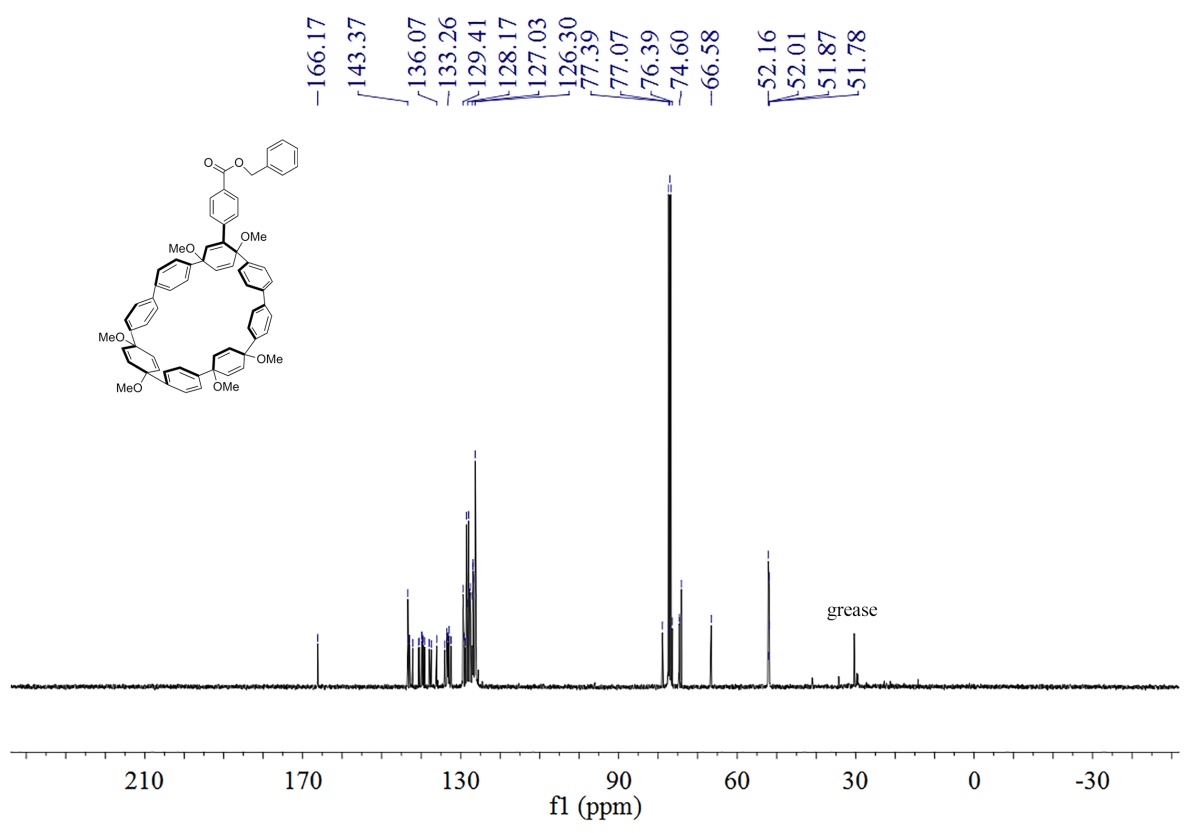


**Fig. S29.** ^13^C NMR spectrum of (4-Benzyloxycarbonylphenyl)- substituted macrocycle **11**.


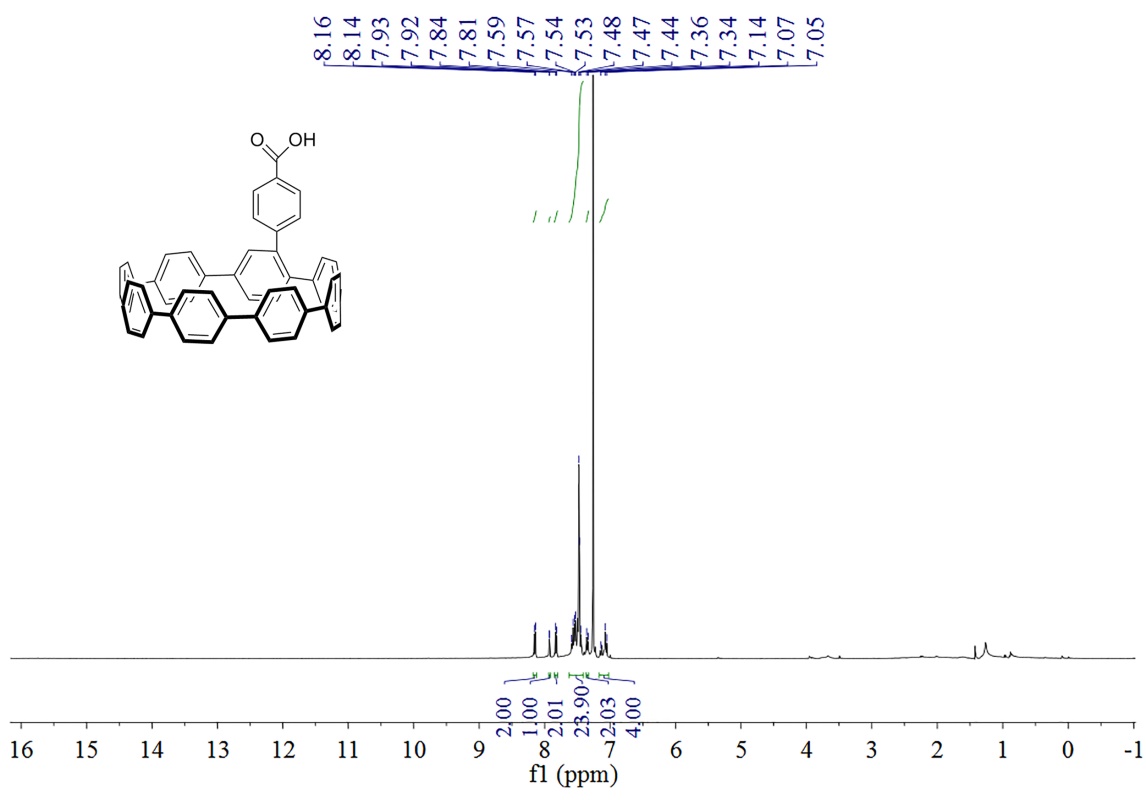


**Fig. S30.** ^1^H NMR spectrum of [8]CPP-COOH **4**.


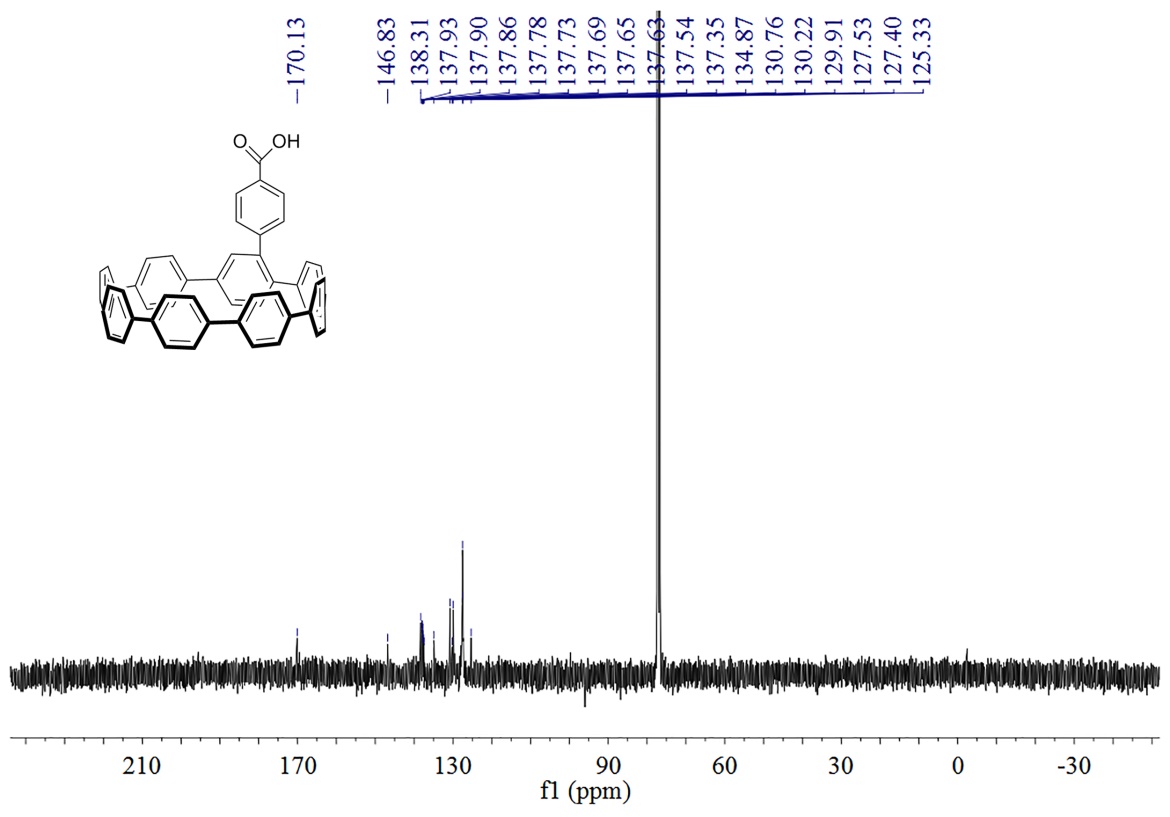


**Fig. S31.** ^13^C NMR spectrum of [8]CPP-COOH **4**.

**4. References**

[1] J. Xia, J.W. Bacon and R. Jasti, "Gram-scale synthesis and crystal structures of [8]- and [10]CPP, and the solid-state structure of C60@[10]CPP," *Chem. Sci.* vol. 3, no. 10, pp. 3018-3021, 2012.

[2] J. Xia, M.R. Golder, M.E. Foster, B.M. Wong and R. Jasti, "Synthesis, characterization, and computational studies of cycloparaphenylene dimers," *J. Am. Chem. Soc.* vol. 134, no. 48, pp. 19709-19715, 2012.

[3] J. Huang, N. Sun, Y. Dong, R. Tang, P. Lu, P. Cai, Q. Li, D. Ma, J. Qin and Z. Li, "Similar or Totally Different: The Control of Conjugation Degree through Minor Structural Modifi cations, and Deep-Blue Aggregation-Induced Emission Luminogens for Non-Doped OLEDs," *Adv. Funct. Mater.* vol. 23, no. 18, pp. 2329–2337, 2013.
